# Reversible Photoswitching of Donor–Acceptor Stenhouse Adducts in Water

**DOI:** 10.1021/jacs.5c19813

**Published:** 2025-12-22

**Authors:** Francisco G. Blandón-Cumbreras, Marek Jurtík, Aneta Závodná, Petr Janovský, Michal Rouchal, Robert Vícha, Uwe Pischel

**Affiliations:** # CIQSO − Center for Research in Sustainable Chemistry and Department of Chemistry, 16743 University of Huelva, Campus de El Carmen s/n, E-21071 Huelva, Spain; § Department of Chemistry, Faculty of Technology, 495963 Tomas Bata University in Zlín, Vavrečkova 5669, 760 01 Zlín, Czech Republic

## Abstract

Adamantane-substituted donor–acceptor Stenhouse adducts (DASA) form highly stable host–guest complexes with cucurbit­[*n*]­urils (*n* = 7, 8) in water. These assemblies show kinetic stabilization of the colored linear form and, remarkably, exhibit reversible T-type photoswitching in water. These so far elusive features for first-generation DASA reveal a strategy for their potential use in biorelevant contexts.

## Graphical abstract

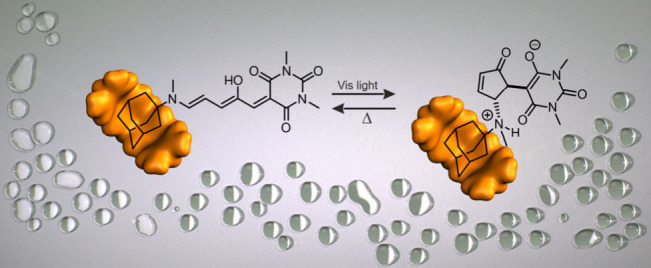

Molecular photoswitches have been in the limelight of the design of stimuli-responsive (supra)­molecular systems. This attraction builds on the spatiotemporal control exerted by light and the diversity of photoswitches. The most popular platforms are based on *E*/*Z* isomerization as found in azobenzenes and hemithioindigos or rely on ring-opening and -closing reactions, typical for spiropyrans, fulgimides or diarylethenes.^1,2^ Donor–acceptor Stenhouse adducts (DASA), whose photochromic properties were first reported in 2014,^3,4^ form part of the latter group. The stable linear form of these photoswitches is colored and is converted by visible-light irradiation into a colorless closed cyclopentenone form (see Scheme 1).^5–7^ In organic solvents, such as toluene, the closed form may revert back to the linear form in a thermal process, known as T-type switching. The colored linear form tends to be more stable in organic media when presenting increased charge-transfer (CT) character, which is typical for first-generation DASA. However, this often penalizes the photoinduced multistep isomerization to the closed form.^8^ To optimize the interplay of electronic/structural factors and molecular properties, various generations of DASA photoswitches were developed by tuning donor and acceptor moieties as well as the triene backbone.^4,9–15^ This molecular diversity has sparked interest in the exploitation of the DASA platform in light-activatable materials,^8,16–18^ photocontrolled phase-transfer,^19^ drug delivery,^20,21^ sensing^22,23^ or photopharmacology.^24^ However, such applications are foremost limited to organic environments (solvents, polymeric materials, *etc*.), while the use of DASA photoswitches in water has been much less successful.^25,26^ The underlying reason is the spontaneous “dark switching” to the closed cyclopentenone form in polar protic media, occurring in the absence of light.^5,27^ Beside other strategies,^28^ it was reported that supramolecular encapsulation of a DASA chromophore could alleviate the undesired “dark switching” in water.^24,29,30^ For example, a cyclodextrin host can stabilize the linear isomer of a second-generation DASA to some extent.^24^ However, rather high concentrations of the photoswitch and host macrocycle as well as acidic pH were required and even then only *ca*. 1% of the linear form was present. In a different approach it was shown that second-generation DASA, containing a tethered amine donor, can be reversibly switched in aqueous medium.^28^ However, a significant amount of organic cosolvent is necessary, i.e., 60 vol% tetrahydrofuran (THF). Hence, the switching of DASA in polar protic solvents remains a challenging task.^8,26,28,31^

**Scheme 1:**
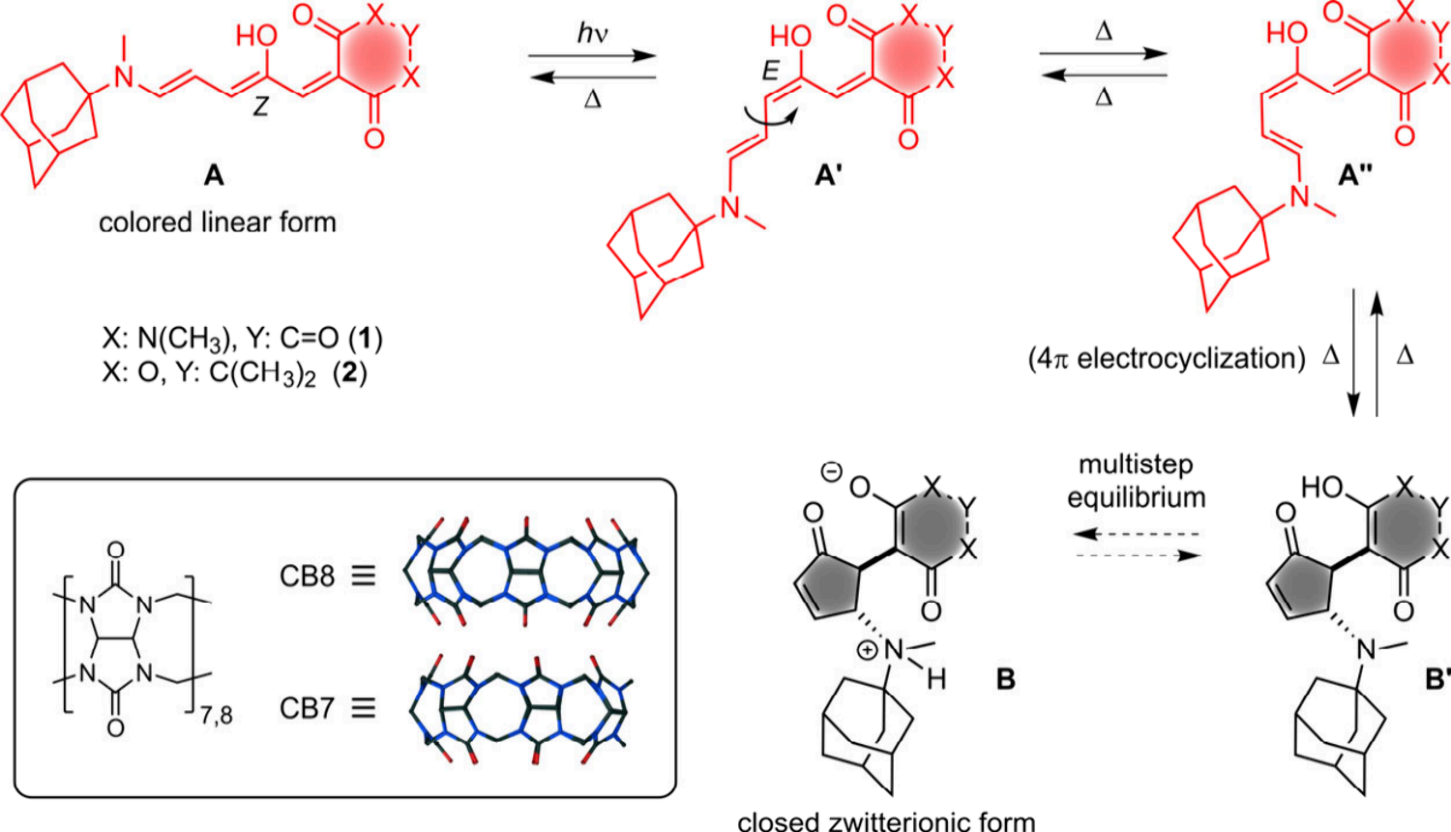
Structures of DASA Photoswitches 1 and 2 and General Switching Mechanism^a^

a The inset shows the structures of the employed CB*n* macrocycles. The mechanistic scheme is partially adapted from ref 26. Copyright 2025 American Chemical Society.

The notion that steric hindrance by voluminous substituents at the amino donor site can disfavor the thermal steps that lead to the formation of the closed cyclopentenone form (Scheme 1) has been experimentally and theoretically cultivated in the literature.^7,13,32^ However, this was not shown so far for DASA in water in the context of avoiding “dark switching”. Being conscious that the synthesis of DASA with sterically overcrowded amino donors is a nontrivial task,^32^ we propose an alternative approach. We hypothesize that the site-selective encapsulation of the amino donor by a strongly binding macrocycle, such as a cucurbit­[*n*]­uril (CB*n*),^33,34^ would exert the desired steric hindrance, while leaving the remaining chromophore in a nonconfined environment. It is noteworthy that cucurbit­[*n*]­urils have already found application in biorelevant contexts.^33–35^

The pronounced CT character of first-generation DASA has been suggested to be the main obstacle for achieving reversible photoswitching in polar protic media.^28^ Herein we demonstrate that significant steric effects can overrule the electronic effects, yielding the recyclable operation of DASA in water.

We designed the first-generation DASA derivatives **1** and **2** (Scheme 1), containing an adamantylamine donor and an acceptor derived from barbituric acid or Meldrum’s acid, respectively (see the Supporting Information for details about the synthesis and analytical characterization, Figures S1–S10). The choice of the adamantylamine was motivated by the generally accepted notion that 1-aminoadamantane forms highly stable host–guest complexes with CB*n* macrocycles (*n* = 7, 8; CB7 and CB8 structures are shown in Scheme 1) at the level of nanomolar to femtomolar affinity (*K ca*. 1.0 × 10^15^ M^–1^ for CB7 and 8.2 × 10^9^ M^–1^ for CB8) in water.^36^ DFT calculations^37,38^ established the adamantane moiety as preferred binding site of CB7 and CB8 for both isomers of **1** and **2**; see Figure 1 for a graphical representation and the Supporting Information (Table S2). The formation of host–guest complexes with CB7 and CB8 was further confirmed by mass spectrometry (Supporting Information, Figures S11–S14) and UV/vis absorption spectroscopy (see below).

**Figure 1:**
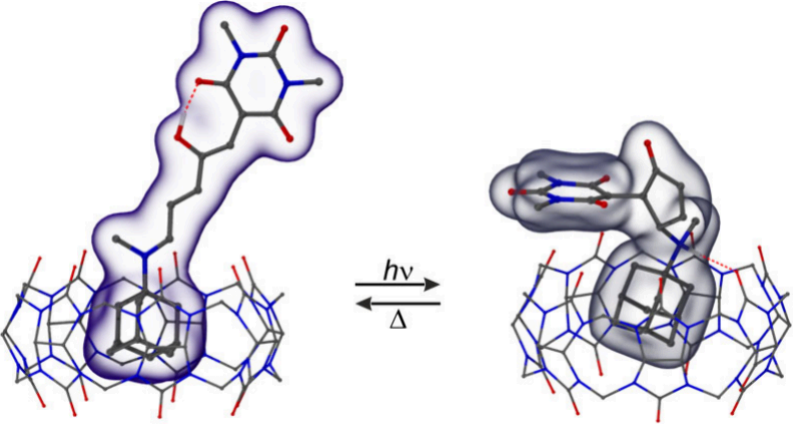
DFT-optimized structures of the CB7 complexes with **1** in the linear (left) and closed form (right).

To demonstrate the CT character of the linear form of **1** and **2**, the UV/vis absorption spectra in solvents of varying polarity were recorded. It is well established that DASA photoswitches show negative solvatochromism and hence, in more polar solvents a blue-shift of the π,π* absorption band is observed.^8,26,39^ The longest-wavelength absorption maximum was plotted against the Dimroth-Reichardt parameter E_T_^N^(30) and the solvatochromic slope was determined, according to reported practice for DASA photoswitches.^40^ For **1** and **2** a slope of –79 nm and –65 nm, respectively, resulted (Supporting Information, Figures S17–S18), which manifests the significant CT nature of the linear form.^40^ Interestingly, the addition of one equivalent CB7 to a solution of **1** or **2** in water produced a blue-shift by about 18–25 nm (Supporting Information, Table S1 and Figures S19 and S20). Likely, this effect is caused by the stabilization of the CT-related partial positive charge at the amine nitrogen atom by ion-dipole interactions with the carbonyl-lined CB7 portal. This observation provides additional hint on the successful complexation of the adamantylamine moiety by the macrocycle. For the larger CB8 macrocycle these effects were also evident, albeit being somewhat less pronounced (blue-shift by 8–9 nm).

To investigate the “dark switching” ^5,27^ of DASA **1** and **2**, the colored linear form was dissolved in THF and then diluted into water, resulting in 10 vol% THF as cosolvent. The half-life (*t*_1/2_) of the linear form of DASA **1** is only about 38 min, as determined by monitoring the disappearance of the longest-wavelength absorption band (Figure 2). For DASA **2** a similarly short *t*_1/2_ of 29 min resulted (Supporting Information, Figure S25). Upon decolorization of the solution, new absorption features between 250 and 300 nm were observed, coincident with the reported spectral signature of the colorless cyclopentenone form.^5^ However, when one equivalent of CB7 or CB8 was present (here no THF as cosolvent was needed), the “dark switching” was significantly altered. Initially *ca*. 25–30% of the dye was present in the linear form (assuming a molar absorption coefficient of *ε* = 110 000 M^–1^ cm^–1^) and the decolorization was considerably slowed down. For example, for DASA **1** a *t*_1/2_ of 12.4 h was determined for the host–guest complex with CB7, corresponding to a stability enhancement by a factor of about 20 (Figure 2). In the case of DASA **2** the effect was even more dramatic, leading to a *t*_1/2_ of 586.3 h and the observation that the supramolecular complex was about 1200 times more stable than the free dye (Supporting Information, Figure S29)! For the larger CB8 macrocycle also considerable benefits for the stabilization of the linear form of **1** and **2** applied, with *t*_1/2_ reaching values of 45.1 and 112.2 h, respectively (Supporting Information, Figures S28 and S30). The significantly slower “dark switching” in the presence of CB*n* is attributed to the increased steric screening around the amino moiety as the principal factor. We presume that the steric overload has its main impact on the C3–C4 bond rotation (A′→A′′) and the 4π electrocyclization (A′′→B′); see Scheme 1.^7,13,32^ In addition, the above-mentioned stabilization of the CT state by ion–dipole interactions with the CB*n* macrocycles could be another contribution to the reduced “dark switching”.

**Figure 2:**
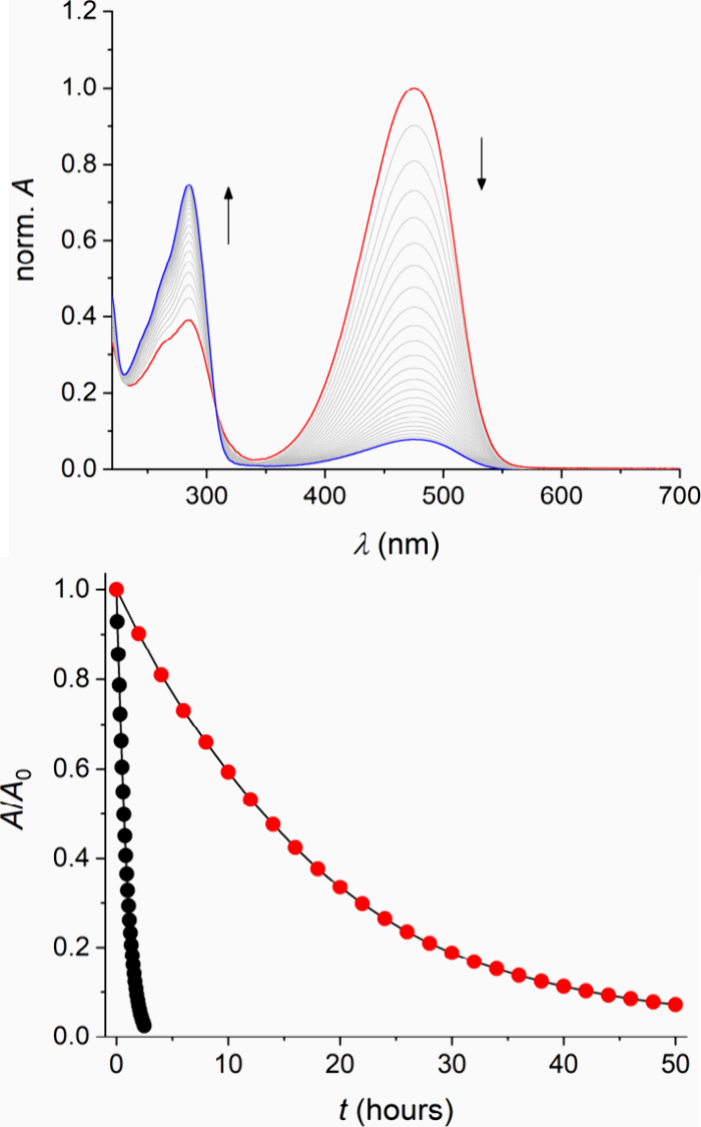
“Dark switching” of DASA **1** in water. Top: Spectral evolution in the presence of one equivalent CB7 (15 μM); the absorption spectra were normalized to 1 at λ_max_. Bottom: Kinetic decays of the colored linear form in the absence (black dots) or presence of CB7 (one equivalent; red dots).

The experimental observations are in very good agreement with theoretical calculations [M06-2X/def2-SVP/CPCM­(water) level of theory] ^41,42^ of the reaction path in the absence and presence of CB7 (Figure 3). For example, for the case of DASA **1** the calculations predict an increase of the activation barrier by about 9.1 kcal mol^–1^ for the A′→A′′ step of the supramolecular complex. For DASA **2**, which experiences even more dramatic stabilization of the linear form, the energy barrier for the A′→A′′ step raises by 11.4 kcal mol^–1^ as compared to the free dye (Figure 3 and Supporting Information, Table S3 and Figures S39–S42). In addition, the energy barrier for the A′′→B′ transformation is raised significantly by 7.4 kcal mol^–1^ for DASA **2** in the presence of CB7.

**Figure 3:**
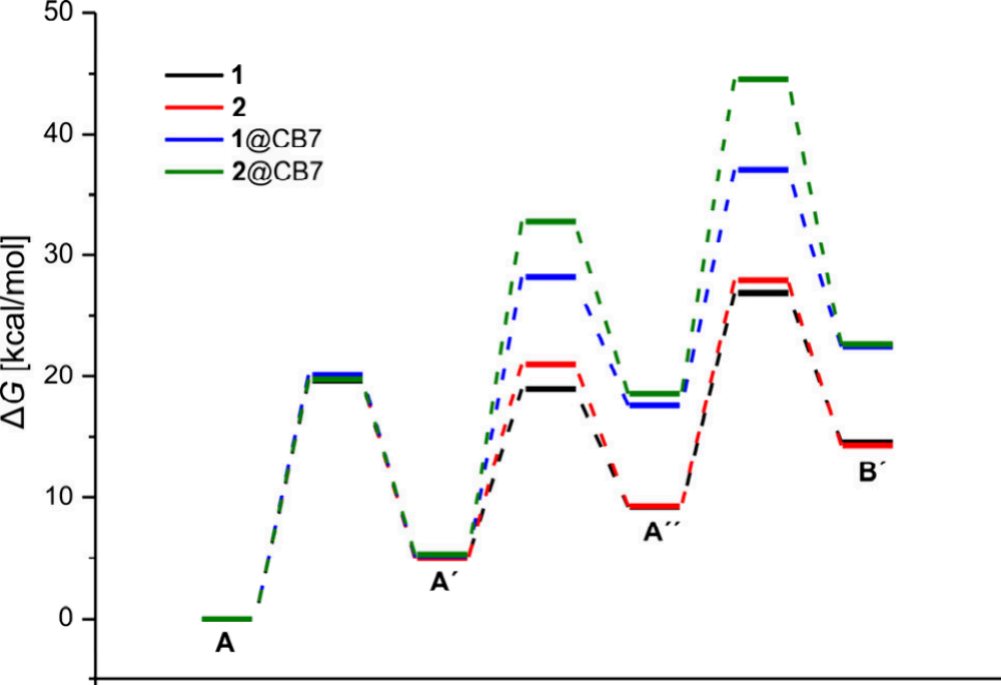
Calculated energy profile for the “dark switching” of DASA **1** and **2** and their CB7 complexes in water.

Next, we investigated the photoswitching of the DASA dyes. Upon irradiation at wavelengths >455 nm, the free dyes showed decolorization, characterized by the disappearance of the π,π* absorption band and the build-up of the closed cyclopentenone isomer with UV absorption below 300 nm (Supporting Information, Figures S26 and S27). It should be noted that the photoinduced ring closing proceeds about 10 times faster than the “dark switching”. The photoreaction of **1** and **2** proceeded with a global quantum yield Φ_r_ of 0.03 and 0.06, respectively. However, for the supramolecular CB7 and CB8 complexes a pronounced drop in the efficiency was observed, being Φ_r_ < 0.005 (Supporting Information, Figures S31–S34). This reflects the increased steric congestion of the host-encapsulated amine donor moiety, interfering in the thermal steps of the mechanism,^7^ akin to the observations for the “dark switching” (see above). However, the photoswitching can be still carried out conveniently on a time scale of a few minutes (see below).

Finally, we sought to achieve reversible T-type photoswitching of the systems. We irradiated the CB7 complex of **1** for time periods of 30–150 s with visible light (>455 nm, at room temperature). For the longest irradiation time a conversion of *ca*. 20% was reached. After switching off the lamp the colored linear form was recovered with a *t*_1/2_ of *ca*. 50 s (Figure 4). For the CB7 complex of DASA **2** the same experiment was performed and a comparable conversion level was observed (Supporting Information, Figure S35). However, the thermal back reaction was not spontaneous at room temperature and heating to 55 °C was necessary to recover the colored linear form. Even then the recovery time was in the order of several minutes. This observation goes along with the considerably higher energetic barrier (difference of 7.2 kcal mol^–1^) for the electrocyclic reversion of the cyclopentenone of **2** as compared to **1** (B′→A′′ in Scheme 1); see the Supporting Information, Table S3. For the CB8 complexes of **1** and **2** a similar switching behavior as for the CB7 complexes was observed (Figure 4 and Supporting Information, Figure S36), including the need to heat to 55 °C in the case of **2**.

**Figure 4:**
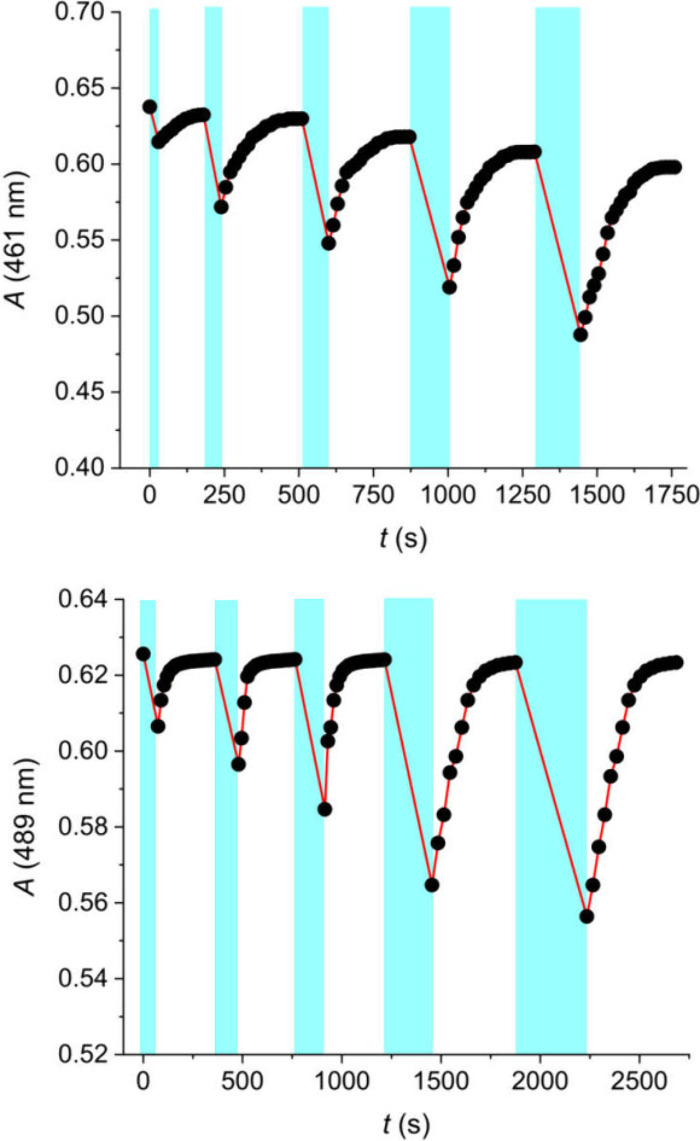
Switching of DASA **1** in the presence of 1 equiv CB7 (top) or CB8 (bottom) in water. Irradiation at >455 nm for variable times (cyan bars; 0.5, 1, 1.5, 2, 2.5 min for CB7 and 1, 2, 2.5, 4, 5 min for CB8) and ring opening at room temperature. The systems show robust recovery over five cycles.

It is noteworthy that for the prolonged irradiation of the CB8 complex of **2** for 600 s about 33% of the linear form was transformed. This surpasses the best performing DASA in aqueous solution, for which *ca*. 20% conversion was observed under comparable conditions.^28^ To compare the switching behavior in water with that of the DASA dyes alone in nonpolar organic medium, experiments in toluene were performed. For both dyes **1** and **2** the expected T-type switching was observed (Supporting Information, Figures S37 and S38). The half-life of the recovery of the linear form is in the order of tens to one hundred seconds (*t*_1/2_ *ca*. 25 s for **1** and 107 s for **2**), that is in the same range as for CB7- or CB8-stabilized DASA **1** in water.

In conclusion, the supramolecular binding of the amine donor of DASA by cucurbit­[*n*]­urils exerts steric effects that favor the stabilization of the colored linear form of first-generation DASA and their reversible photoswitching in water. Importantly, the proposed supramolecular approach is potentially compatible with other macrocycles than the herein employed cucurbit­[*n*]­urils, e.g., persulfonated pillar[6]­arenes that form ultrastable host–guest complexes with adamantane binding motifs.^43^

## Supplementary Material





## Notes

Experimental section, synthesis and characterization of DASA **1** and DASA **2**, mass spectra of host–guest complexes, additional data on “dark switching” and reversible photoswitching of DASA dyes and their host–guest complexes with CB7 and CB8, fluorescence emission of DASA host–guest complexes with CB7 and CB8, and details about theoretical calculations (PDF)
Atomic coordinates of all calculated structures (TXT)
