# Supplementary material for: Reversible Photoswitching of Donor–Acceptor Stenhouse Adducts in Water
Source: J Am Chem Soc. 2025 Dec 22;148(1):130–4. doi: 10.1021/jacs.5c19813 (PMC12814171; doi:10.1021/jacs.5c19813)
Supplement: Supplementary file 1 [file ja5c19813_si_001.pdf]

SUPPORTING INFORMATION

Reversible Photoswitching of Donor–Acceptor Stenhouse Adducts in Water

Francisco G. Blandón-Cumbreras,[a] Marek Jurtík,[b] Aneta Závodná,[b] Petr Janovský,[b] Michal Rouchal,*[,b] Robert Vícha,[b] and Uwe Pischel*[,a]

[a] *CIQSO – Center for Research in Sustainable Chemistry and Department of Chemistry, University of Huelva, Campus de El Carmen s/n, E-21071 Huelva, Spain*

Email: uwe.pischel@diq.uhu.es

[b] *Department of Chemistry, Faculty of Technology, Tomas Bata University in Zlín, Vavrečkova 5669, 760 01 Zlín, Czech Republic*

Email: rouchal@utb.cz

Table of contents

1. General methods and materials S3

2. Synthetic procedures S6

3. NMR and HRMS data S10

4. ESI-QTOF-MS of the DASA host–guest complexes with CB7 and CB8 S18

5. UV/vis absorption spectroscopy S22

6. Fluorescence emission of DASA **1** and DASA **2** S25

7. “Dark switching” and photoswitching of DASA **1** and DASA **2** – additional data S28

8. Reversible photoswitching of DASA **2** in the presence of CB7 and CB8 in water S33

9. Photoswitching of DASA **1** and DASA **2** in toluene S34

10. Theoretical calculations S35

11. References S46

1. General methods and materials

Synthesis and characterization

All reagents and solvents for the synthesis were commercially available from Sigma-Aldrich or BLDpharm and used as received without further purification. Analytical TLC was performed with aluminum sheets pre-coated with silica gel 60 F_{254} (Merck). The ^{1}H, ^{13}C and HSQC NMR spectra were obtained on a Bruker Avance 400 MHz or 500 MHz HPPR2. ^{1}H and ^{13}C NMR chemical shifts were referenced to the signal of the residual solvent [$CDCl_3$, ^{1}H: $\delta = 7.26$ ppm; ^{13}C: $\delta = 77.23$ ppm]. The signal multiplicity is indicated by ‘s‘ for singlet, ‘d‘ for doublet, ‘t‘ for triplet, and ‘m‘ for multiplet. High-resolution mass spectra were obtained on a quadrupole time-of-flight mass spectrometer (6530 Q-TOF, Agilent Technologies, Santa Clara, USA) equipped with an electrospray ionization source (ESI). All experiments were performed in the positive and negative-ion polarity mode. Individual samples (with concentrations of 7.5 μM) of the dyes were infused into the ESI source as acetonitrile (ACN) solutions using a syringe pump with a constant flow rate of 3 $\mu l \cdot min^{-1}$. An equimolar mixture of DASA **1** or **2** and CB7 or CB8 (with concentration of 15 μM) was infused into the ESI source as water solution using a syringe pump with a constant flow rate of 3 $\mu l \cdot min^{-1}$. The mass spectrometer was operated with the following parameters: a capillary voltage of ±4.0 kV, a nebulizer pressure of 275.79 kPa, a drying gas flow rate of 8.0 L min^{-1}, and a drying gas temperature of 300 °C. Mass spectra were acquired over the *m/z* 50–3000 range at a scan rate of 3 scan s^{-1}. Accurate mass measurements were obtained by using a calibrating solution, involving internal reference masses (purine ($C_5H_4N_4$) at *m/z* 121.050873, and HP-0921 [hexakis-(1*H*,1*H*,3*H*-tetrafluoropentoxy)-phosphazene] ($C_{18}H_{18}O_6N_3P_3F_{24}$) at *m/z* 922.009798).

Characterization of DASA – dark switching and photoinduced ring closing

The measurements were done at room temperature (23 ºC) with air-equilibrated solutions contained in quartz cuvettes with 1 cm optical pathlength. Water, which was used for the spectroscopic measurements, was of Milli-Q® purity (EQ 7000 ultrapure water system).

To determine the reaction rate of the "dark switching" process of DASA **1** and **2** in aqueous media, tetrahydrofuran (THF) was employed as co-solvent. This allowed to minimize the time of dissolving the dyes, thereby avoiding excessive "dark switching" during the sample preparation. Stock solutions of the photoswitches **1** and **2** (0.25 mM in THF) were prepared. Subsequently, 300 µL of each stock solution was added to 2700 µL of Milli-Q grade water in a quartz cuvette. After rapid mixing to ensure homogeneity, the dark switching process was monitored using an Agilent CARY 5000 UV-Vis spectrophotometer in kinetic mode.

Solutions of the host–guest complexes of **1**or **2** with CB7 or CB8 were prepared as follows. Stock solutions of CB7 (0.1 mM) or CB8 (0.089 mM) in water were added to a pre-weighed amount of solid photoswitch **1** or **2** in a 1:1 molar ratio. The mixtures were sonicated in an ultrasonic bath for 15 minutes to ensure complete dissolution and complex formation. Immediately after preparation, the "dark switching" process was monitored using a CARY 5000 spectrophotometer from Agilent Technologies.

For the photoinduced ring closing experiments a 150 W xenon lamp (Oriel GmbH & Co. KG) with a 455-nm long-pass filter or a 440-nm band-pass filter were employed. The progression of the photochemical reactions was followed by UV/vis absorption measurements with a CARY 5000 spectrophotometer from Agilent Technologies. The quantum yield of the DASA ring closing was determined by employing the initial-rate method.[1] For this the colored linear form was irradiated at 440

nm (band-pass filter) and potassium tri-oxalatoferrate(III)trihydrate was used as actinometer (Φ_r = 1.01 in a buffered solution of 0.23 M CH_3COONa/0.05 M H_2SO_4).[2] The molar absorption coefficient of the colored linear form of DASA **1** or **2** in water was approximated to the value measured in tetrahydrofuran (ε = 110000 $M^{-1}cm^{-1}$).

The thermally-activated back conversion of the closed to the colored linear form of DASA **1** was conducted using the following procedure. The UV/vis absorption spectrum of a 25 µM solution of **1**@CB*n* (*n* = 7 or 8) was initially recorded. Then, the cuvette containing the solution was irradiated for a given time. After cessation of the irradiation the recovery of the colored linear form was recorded in the kinetic mode of the CARY 5000 spectrophotometer. This procedure was repeated for a total of five cycles.

In the case of DASA **2** the experiment was conducted as follows. The UV/vis absorption spectrum of a 25 µM solution of **2**@CB*n* (*n* = 7 or 8) was initially recorded. The cuvette containing the solution was then irradiated, and the UV/vis absorption spectrum was subsequently measured. The solution was then transferred to a glass vial and heated in a water bath at 55 ºC. After 5 minutes of heating, the liquid was reintroduced into the cuvette, and the absorbance was measured at λ_{max}. This process was repeated until the absorbance returned to its pre-irradiation level. The entire procedure was repeated for a total of five cycles.

2. Synthetic procedures

5-(Furan-2-ylmethylidene)-1,3-dimethylpyrimidine-2,4,6(1*H*,3*H*,5*H*)-trione (1')

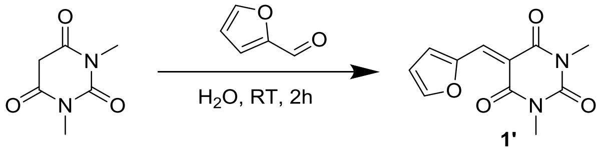

1,3-Dimethylpyrimidine-2,4,6(1*H*,3*H*,5*H*)-trione (1.56 g, 10 mmol) and 2-furaldehyde (961 mg, 10 mmol) were added to 40 mL water and the mixture was stirred at room temperature for 2 hours. During the course of the reaction a yellow precipitate formed. The precipitated solid was collected by vacuum filtration and washed twice with 30 mL cold water. The collected solid was dissolved in dichloromethane (75 mL) and washed with 30 mL saturated aqueous $NaHSO_3$, 30 mL water, 30 mL saturated aqueous $NaHCO_3$, and 30 mL brine. The organic layer was dried over $MgSO_4$, filtered, and the solvent was removed by rotary evaporation to give 2.26 g (96% yield) of the product as a bright-yellow powder. The ^{1}H NMR spectroscopic data were found to match the previously reported values.[3]

DASA 1

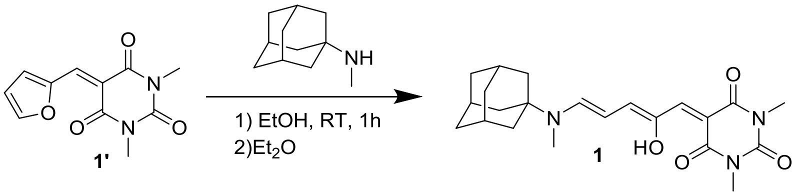

5-(Furan-2-ylmethylidene)-1,3-dimethylpyrimidine-2,4,6(1*H*,3*H*,5*H*)-trione (187 mg, 0.9 mmol) was suspended in 10 mL of ethanol and *N*-methyladamantylamine (150 mg, 0.9 mmol) was added. The mixture was stirred at room temperature for 2 hours, followed by the addition of an excess of diethylether. The reaction mixture was then filtered to collect the precipitated solid (unreacted **1**'). The filtered liquid was dried under vacuum, triturated with water, filtered, and washed several times with cold diethyl ether to afford the product as a purple solid (300 mg, 90% yield).

^{1}H NMR (500 MHz, $CDCl_3$) δ 12.56 (s, 1H), 7.55 (d, J = 12.0 Hz, 1H), 7.11 (s, 1H), 6.79 (d, J = 12.0 Hz, 1H), 6.13 (t, J = 12.0 Hz, 1H), 3.36 (s, 3H), 3.35 (s, 3H), 3.10 (s, 3H), 2.28 (s, 3H), 1.91 (s, 6H), 1.78 (m, 3H), 1.67 (m, 3H). **^{13}C NMR** (126 MHz, $CDCl_3$) δ 165.20, 163.57, 153.91, 152.18, 151.88, 146.74, 138.47, 103.52, 98.15, 61.62, 40.85, 35.74, 32.01, 29.53, 28.54, 28.38. **HRMS** (*m*/*z*) positive: calculated for $C_{22}H_{30}N_3O_4^+$: 400.2231 [M+H$^+$]$^+$; found: 400.2223.

5-(Furan-2-ylmethylene)-2,2-dimethyl-1,3-dioxane-4,6-dione (2')

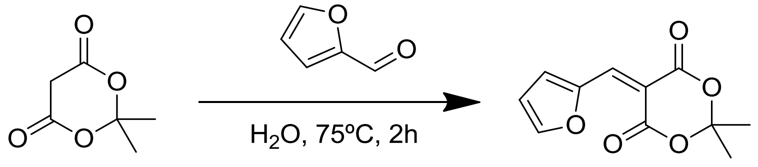

2'

2,2-Dimethyl-1,3-dioxane-4,6-dione (3.00 g, 20 mmol) and 2-furaldehyde (1.92 g, 20 mmol) were added to 40 mL water. The mixture was stirred and heated to 75 ºC for 2 h. During the reaction a yellow solid formed. The precipitate was collected by vacuum filtration and washed twice with 40 mL cold water. Then the solid was re-dissolved in dichloromethane, washed with 40 mL saturated aqueous $NaHSO_3$, 40 mL water, and 40 mL brine. The organic layer was dried over Na_2SO_4 anhydrous, filtered and the volatiles were removed under reduced pressure to yield 4.3 g (98% yield) of 5-(furan-2-ylmethylene)-2,2-dimethyl-1,3-dioxane-4,6-dione as a bright yellow powder. All ^{1}H NMR spectroscopic data were found to match the previously reported values.[4]

DASA 2

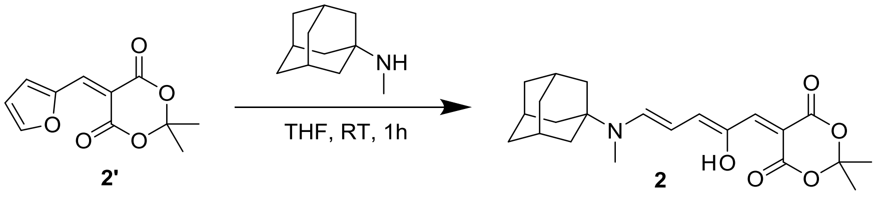

5-(Furan-2-ylmethylene)-2,2-dimethyl-1,3-dioxane-4,6-dione (269 mg, 1.2 mmol) was dissolved in 10 mL of dry tetrahydrofuran. To this dispersion *N*-methyladamantylamine (200 mg, 1.2 mmol) was added. The mixture was stirred at room temperature for 30 minutes, followed by cooling to 0 ºC for 30 min. The reaction mixture was then filtered to collect the precipitated solid. The solid was washed with cold diethyl ether and vacuum-dried to afford the product as a purple solid (400 mg, 85% yield).

^{1}H NMR (400 MHz, $CDCl_3$) δ 11.44 (s, 1H), 7.53 (d, *J* = 12.0, 1H), 7.06 (s, 1H), 6.74 (d, *J* = 12.0 Hz, 1H), 6.09 (t, *J*=12.0 Hz, 1H), 3.08 (s, 1H), 2.28 (s, 3H), 1.92 (s, 6H), 1.78 (m, 3H), 1.71 (s, 6H), 1.68 (m, 3H). **^{13}C NMR** (101 MHz, $CDCl_3$) δ 167.11, 165.30, 153.77, 151.52, 145.02, 138.94, 103.36, 102.67, 90.41, 61.44, 40.71, 35.61, 31.82, 29.41, 26.69. **HRMS** (*m*/*z*) positive: calculated for $C_{22}H_{30}NO_5^+$: 388.2118 $[M+H^+]^+$; found: 388.2106

3. NMR and HRMS data

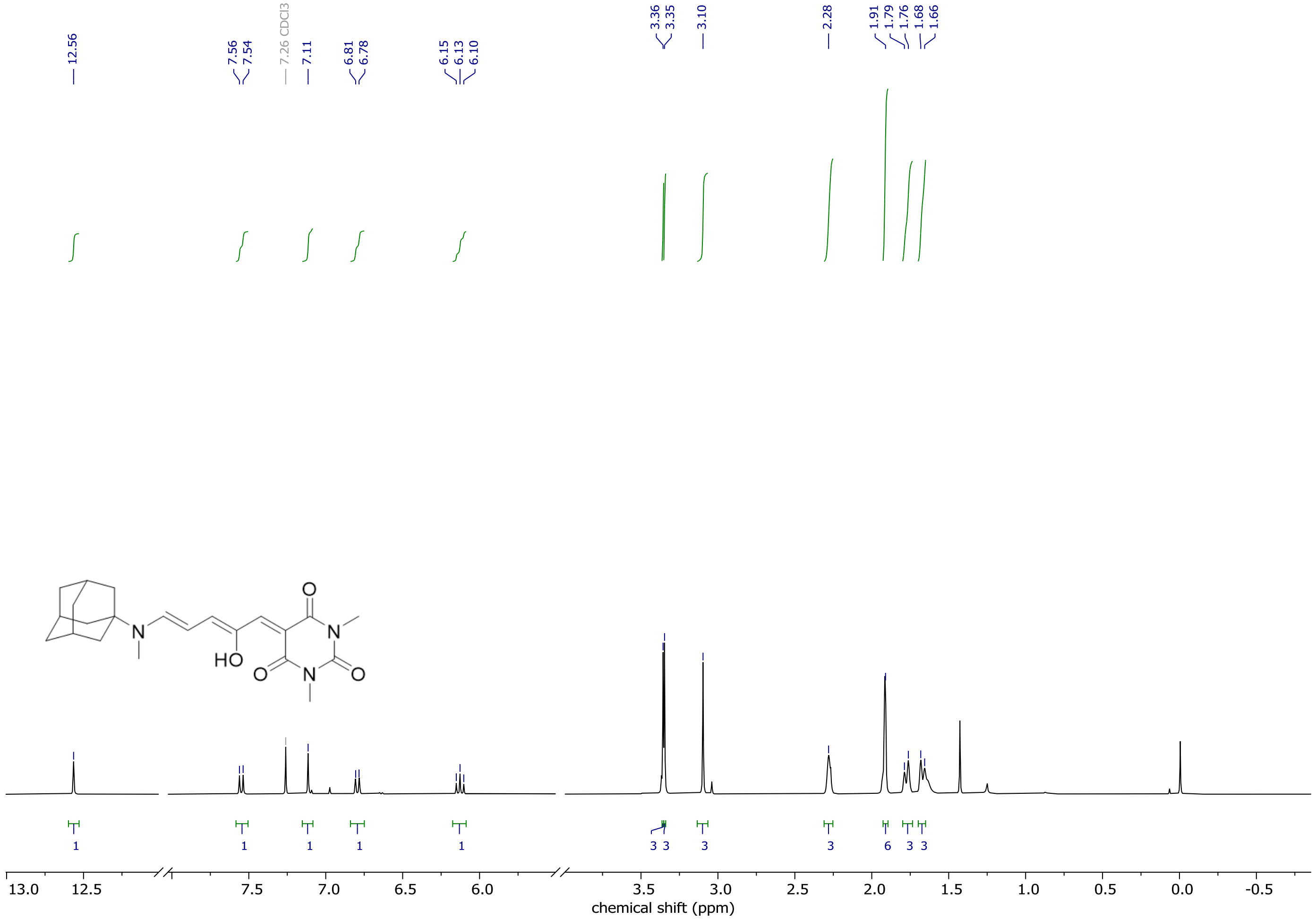

Figure S1. ^{1}H NMR spectrum (500 MHz) of DASA **1** in $CDCl_3$.

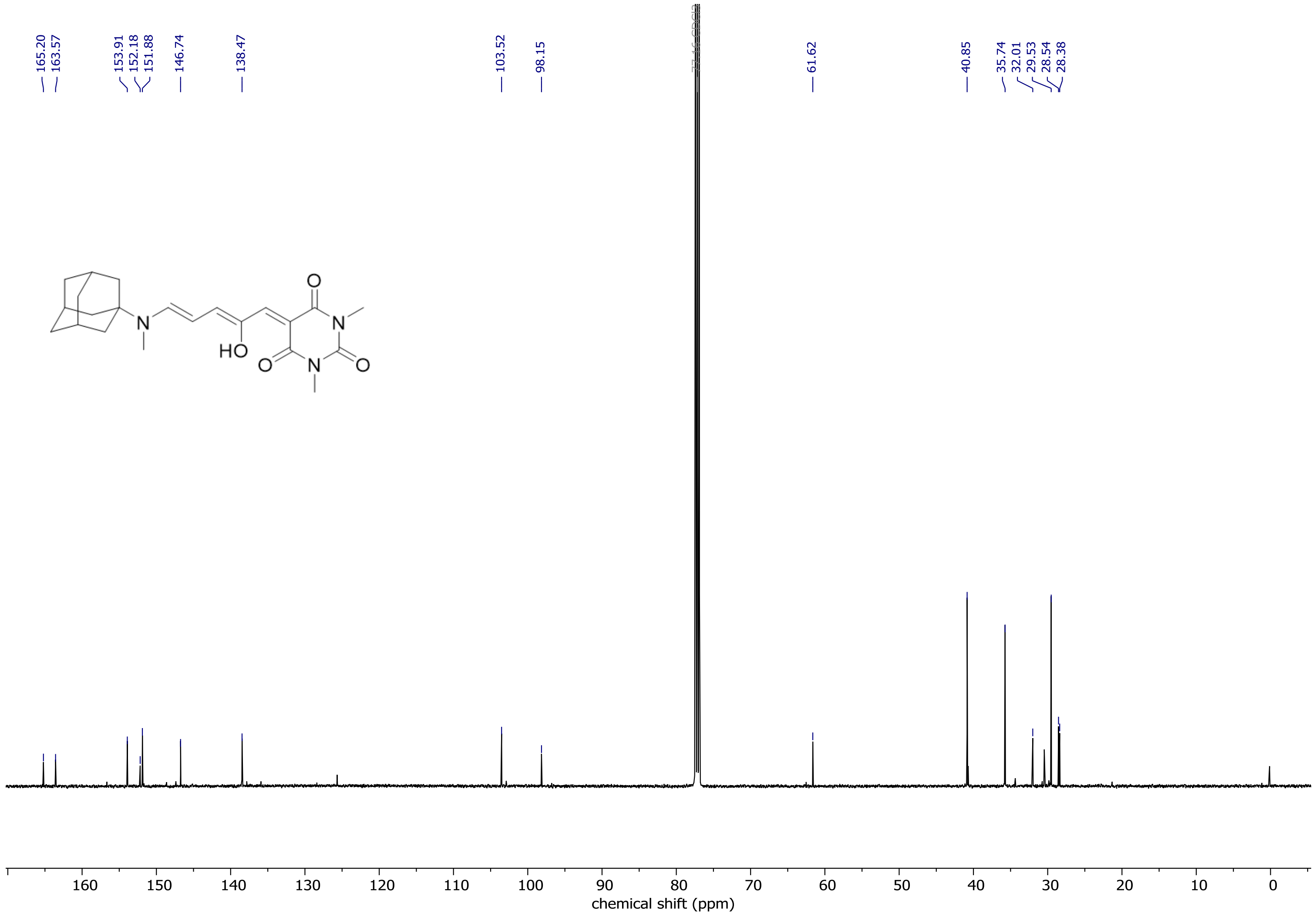

Figure S2. ^{13}C NMR spectrum (126 MHz) of DASA **1** in $CDCl_3$.

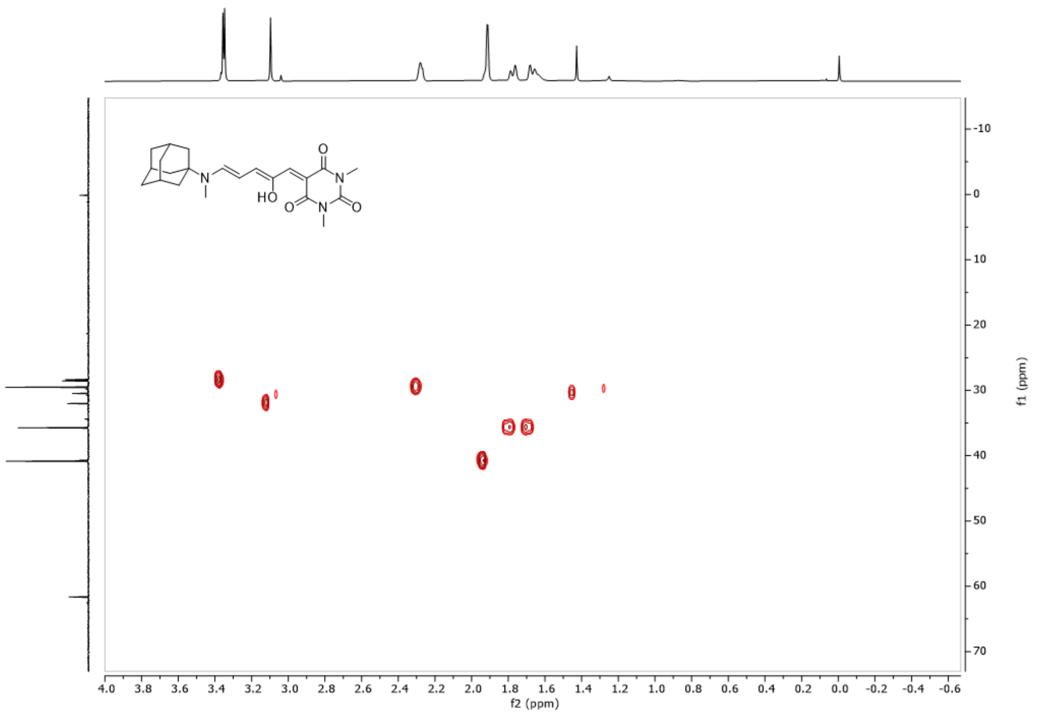

Figure S3. Partial ^{1}H–^{13}C HSQC spectrum (500 MHz) of DASA **1** in $CDCl_3$.

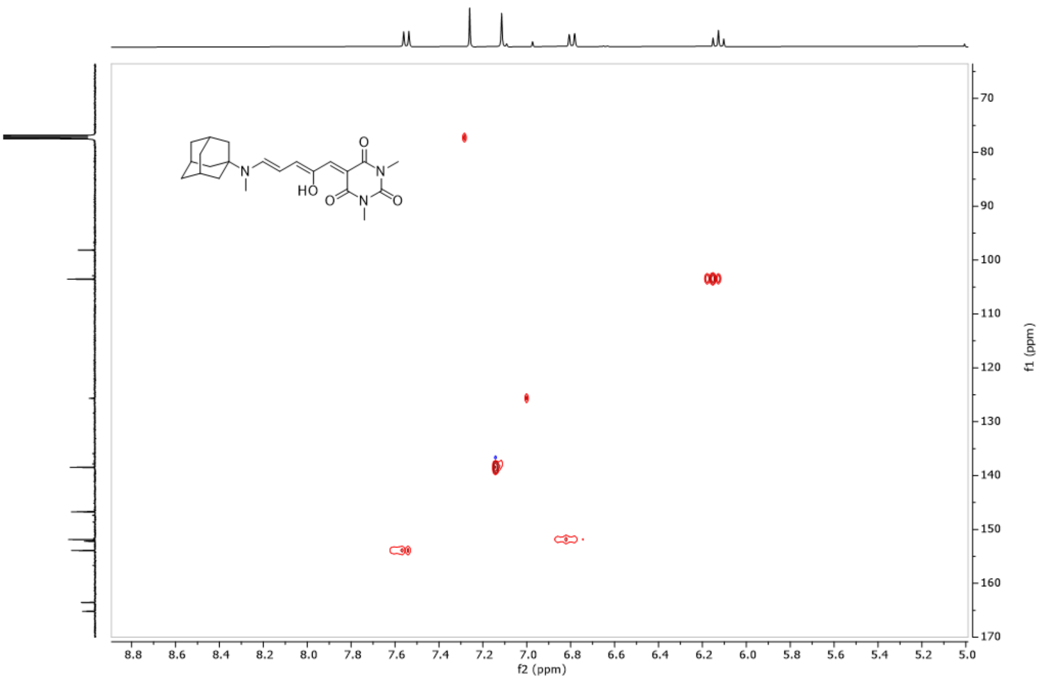

Figure S4. Partial ^{1}H–^{13}C HSQC spectrum (500 MHz) of DASA **1** in $CDCl_3$.

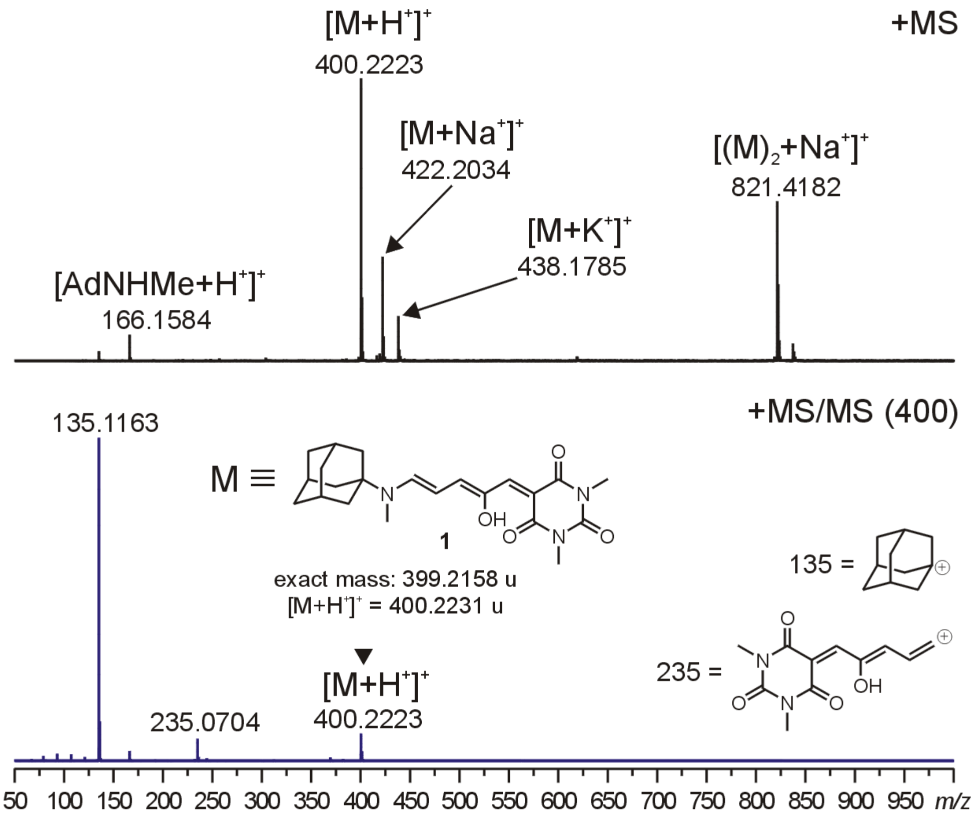

Figure S5. The positive-ion ESI-QTOF-MS (top) and MS/MS (bottom) of an acetonitrile solution of DASA **1**. The assignments for the observed signals are shown in square brackets. The fragmented ion in MS/MS is marked with a downward-facing triangle.

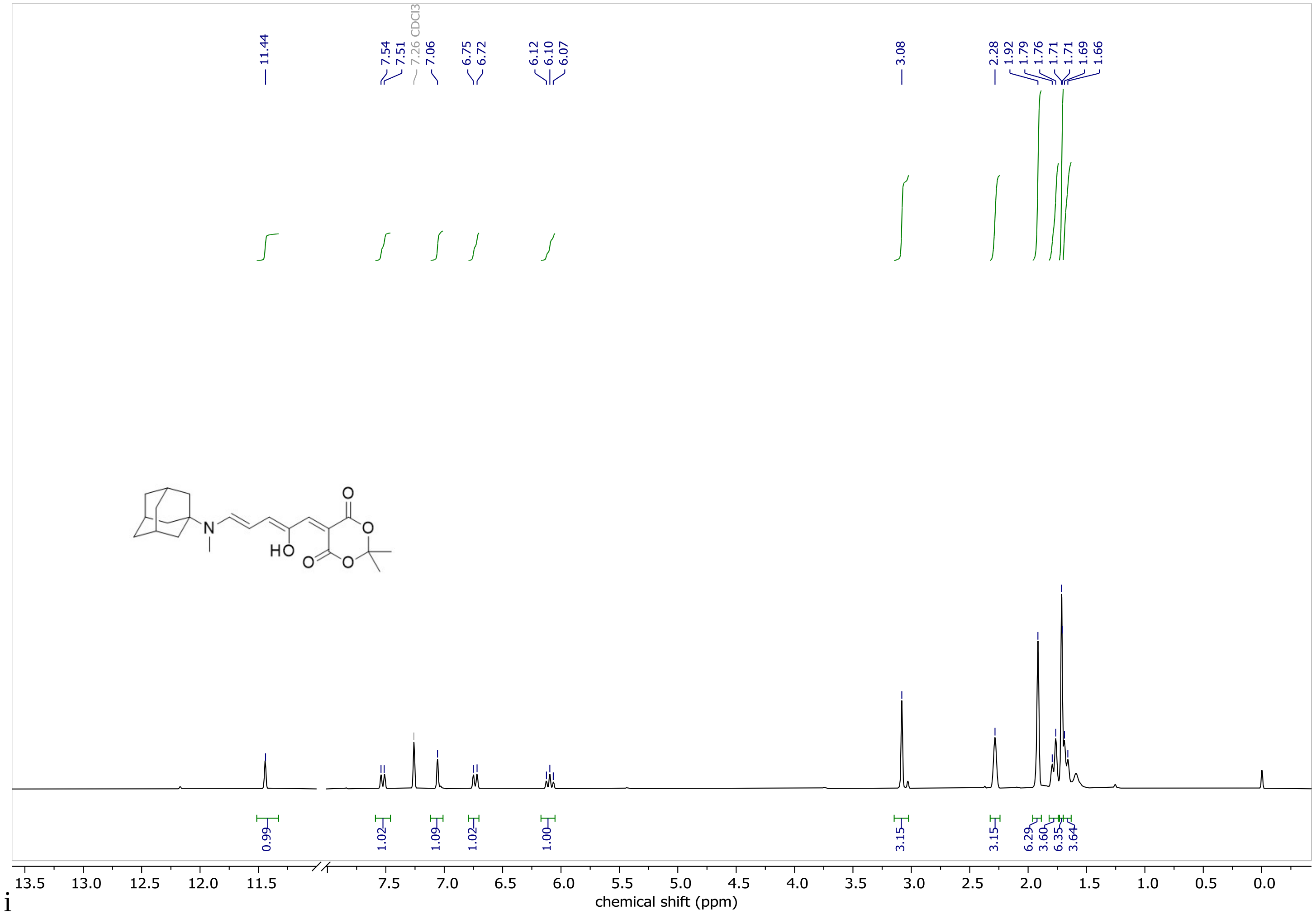

i

Figure S6. ^{1}H NMR spectrum (400 MHz) of DASA **2** in $CDCl_3$.

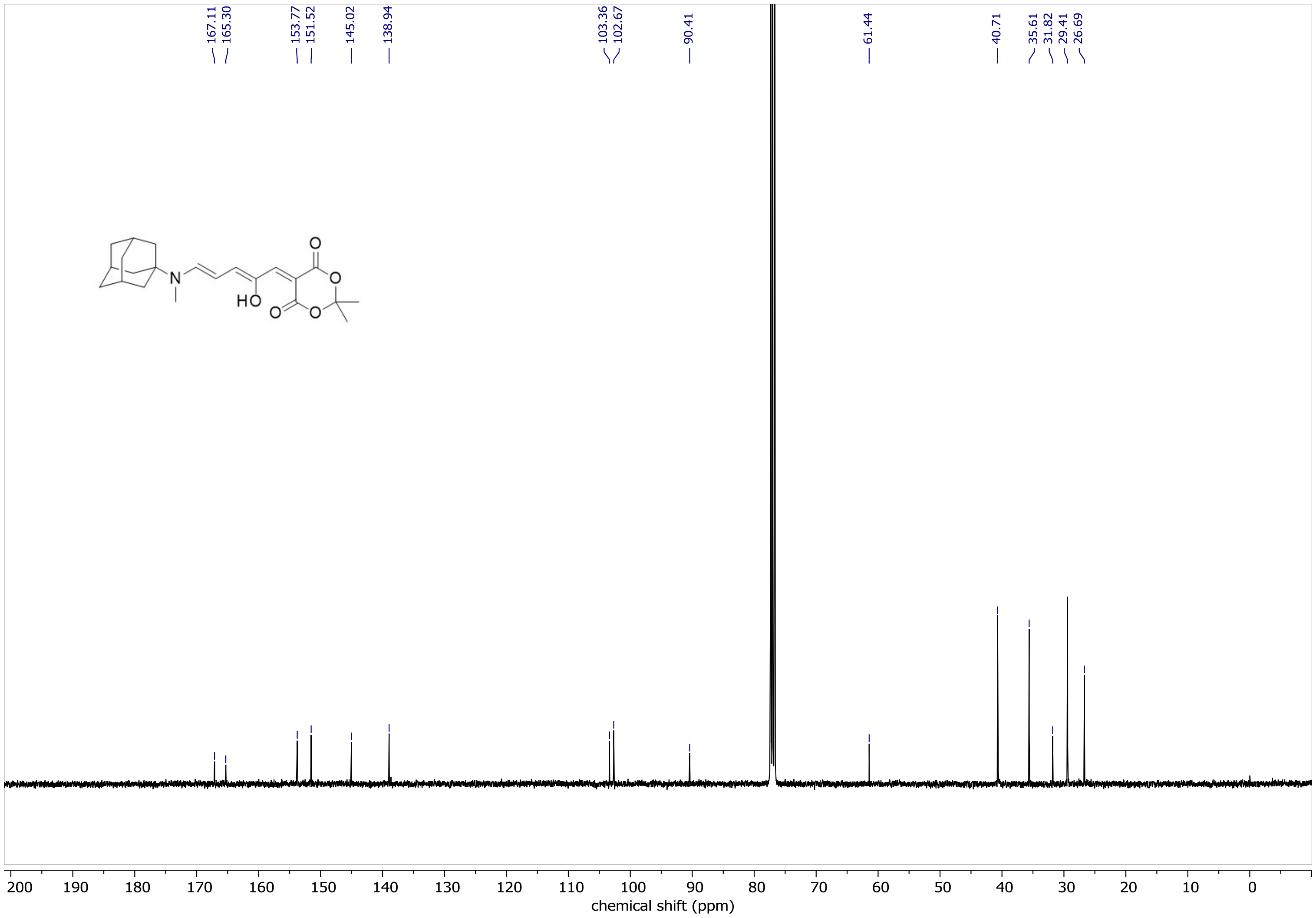

Figure S7. ^{13}C NMR spectrum (101 MHz) of DASA **2** in $CDCl_3$.

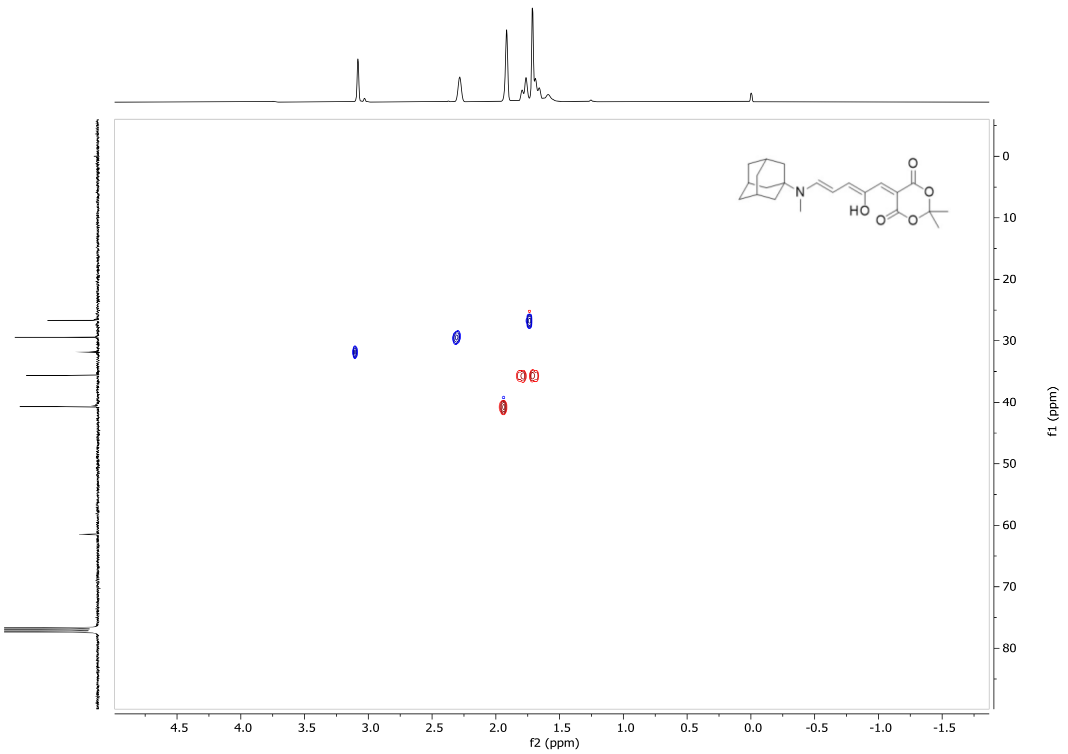

Figure S8. Partial ^{1}H–^{13}C HSQC spectrum (500 MHz) of DASA **2** in $CDCl_3$.

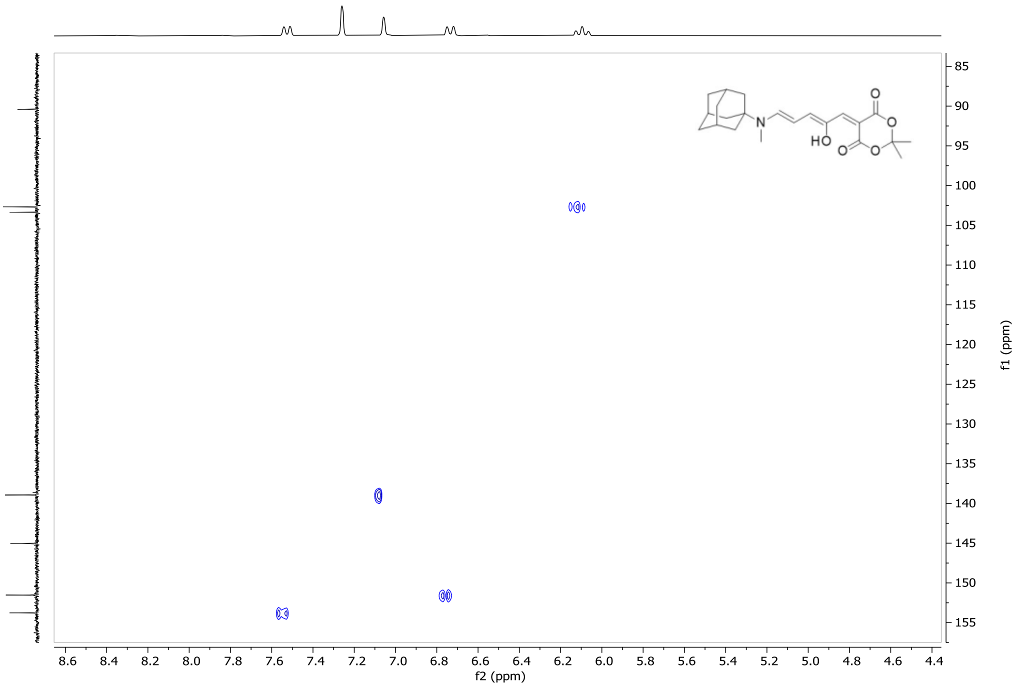

Figure S9. Partial ^{1}H–^{13}C HSQC spectrum (500 MHz) of DASA **2** in $CDCl_3$.

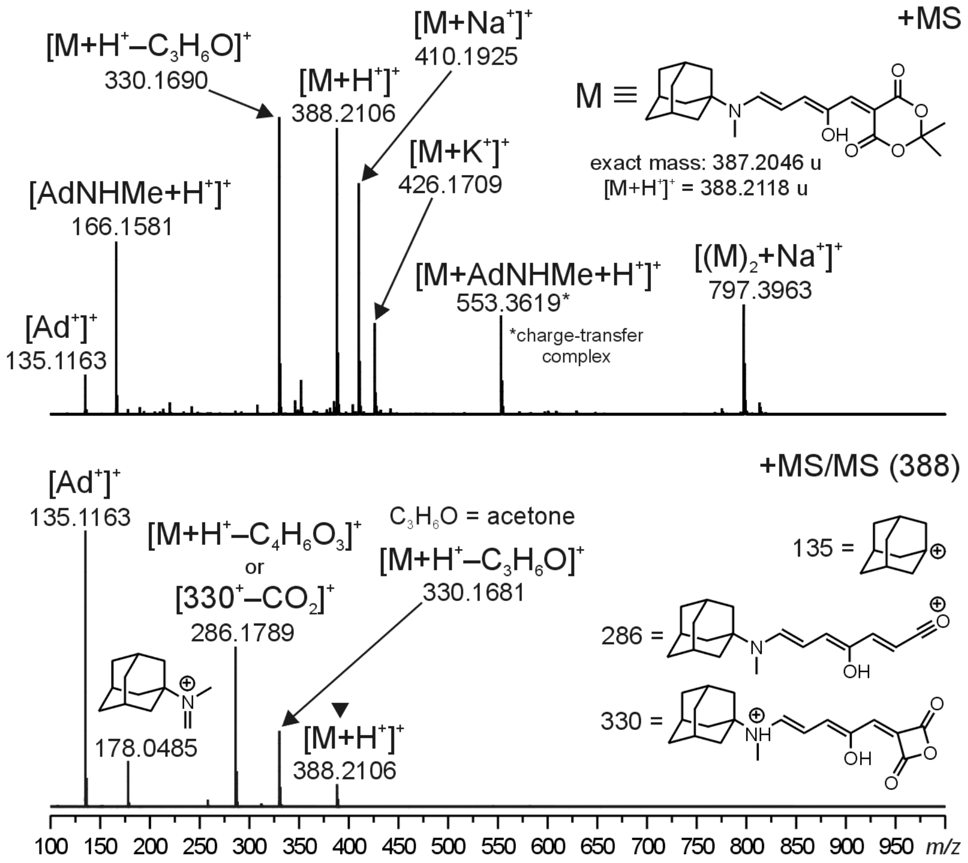

Figure S10. The positive-ion ESI-QTOF-MS (top) and MS/MS (bottom) of an acetonitrile solution of DASA **2**. The assignments for the observed signals are shown in square brackets. The fragmented ion in MS/MS is marked with a downward-facing triangle.

4. ESI-QTOF-MS of the DASA host–guest complexes with CB7 and CB8

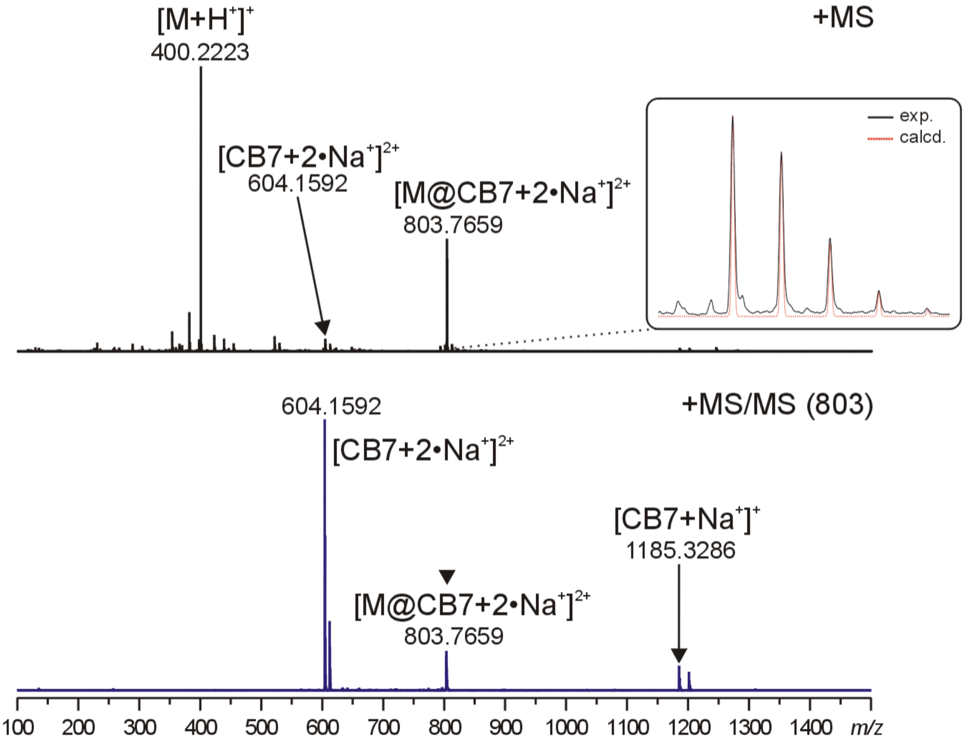

Figure S11. The positive-ion ESI-QTOF-MS (top) and MS/MS (bottom) of an aqueous solution of DASA **1** and CB7. The experimental and calculated isotopic patterns for the ion 803 *m/z* are shown in the inset (top). The assignments for observed signals are shown in square brackets. The fragmented ion in MS/MS is marked with a downward-facing triangle.

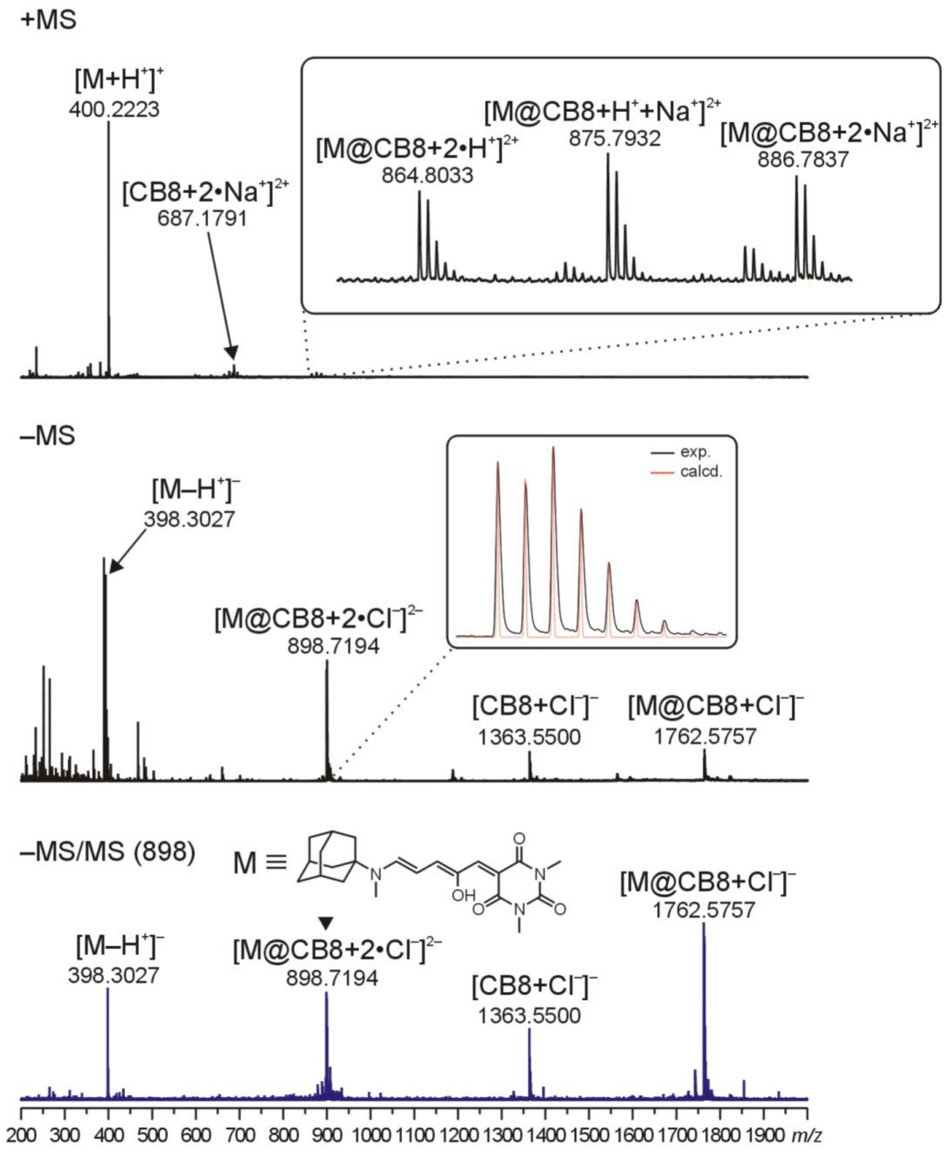

Figure S12. The positive-ion ESI-QTOF-MS (top), negative-ion ESI-QTOF-MS (middle), and negative-ion MS/MS (bottom) of an aqueous solution of DASA **1** and CB8. The experimental and calculated isotopic patterns for the ion 898 *m/z* are shown in the inset (middle). The assignments for observed signals are shown in square brackets. The fragmented ion in MS/MS is marked with a downward-facing triangle.

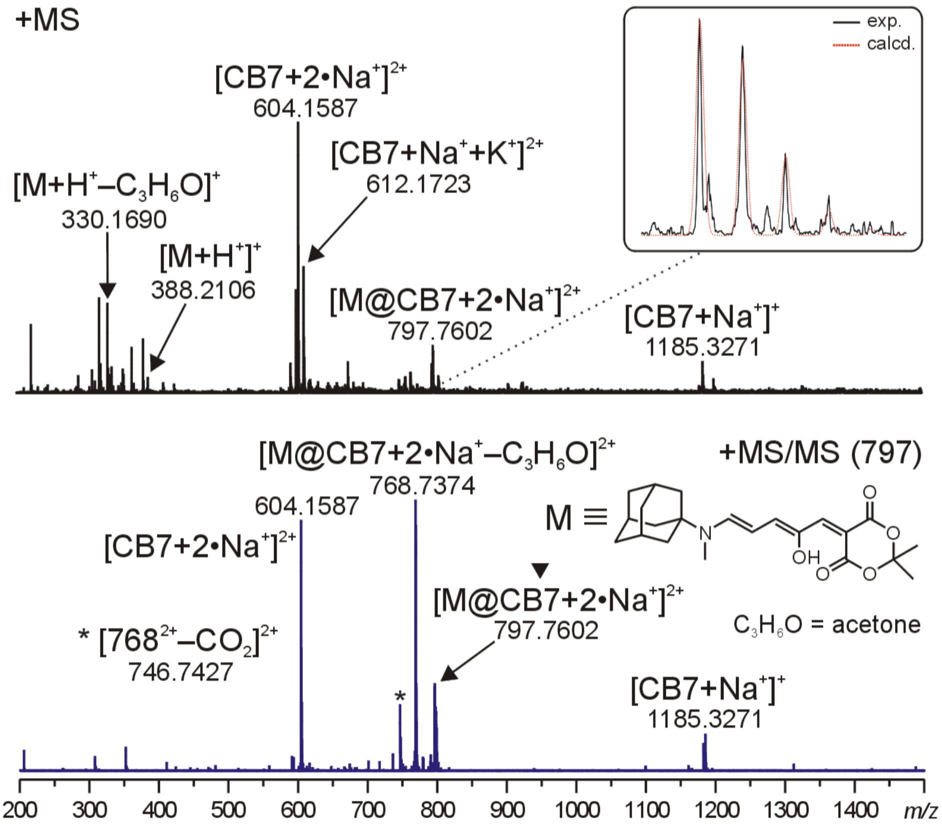

Figure S13. The positive-ion ESI-QTOF-MS (top) and MS/MS (bottom) of an aqueous solution of DASA **2** and CB7. The experimental and calculated isotopic patterns for the ion 797 *m/z* are shown in the inset (top). The assignments for observed signals are shown in square brackets. The fragmented ion in MS/MS is marked with a downward-facing triangle.

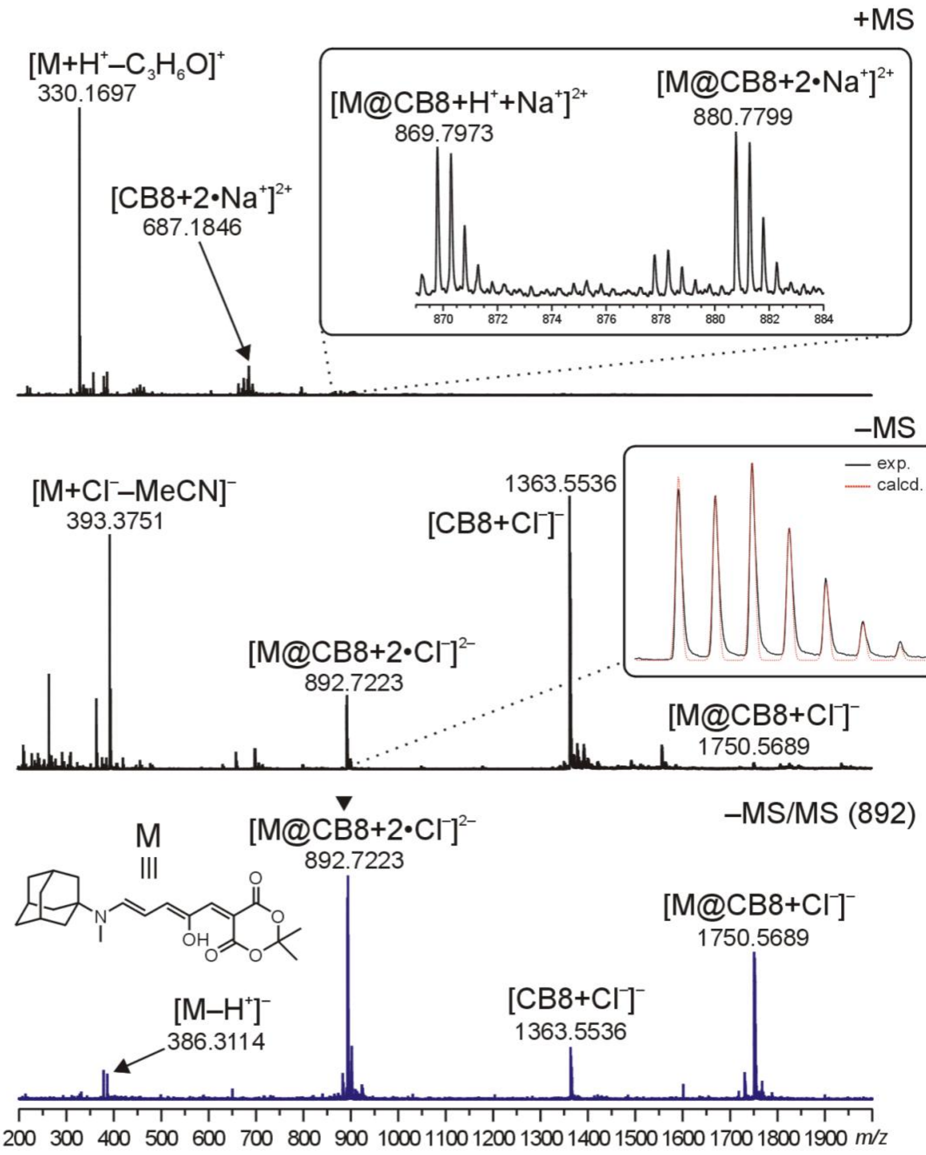

Figure S14. The positive-ion ESI-QTOF-MS (top), negative-ion ESI-QTOF-MS (middle), and negative-ion MS/MS (bottom) of an aqueous solution of DASA **2** and CB8. The experimental and calculated isotopic patterns for the ion 892 *m/z* are shown in the inset (middle). The assignments for observed signals are shown in square brackets. The fragmented ion in MS/MS is marked with a downward-facing triangle.

5. UV/vis absorption spectroscopy

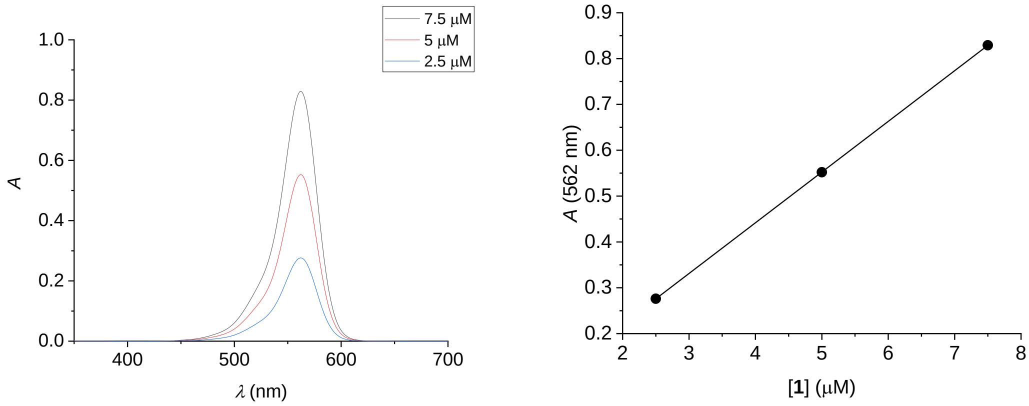

Figure S15. UV/vis absorption spectra of DASA **1** at varying concentrations (left) and corresponding Lambert-Beer plot (right). $\varepsilon = 1.1 \times 10^5$ M^{-1} cm^{-1}.

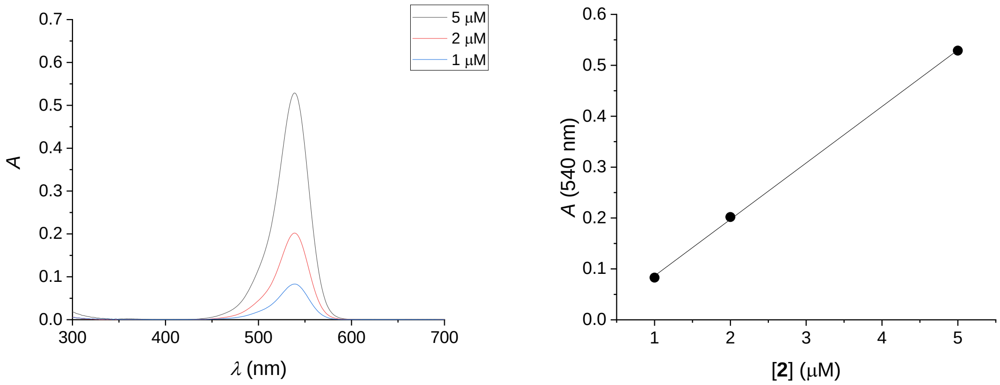

Figure S16. UV/vis absorption spectra of DASA **2** at varying concentrations (left) and corresponding Lambert-Beer plot (right). $\varepsilon = 1.1 \times 10^5$ M^{-1} cm^{-1}.

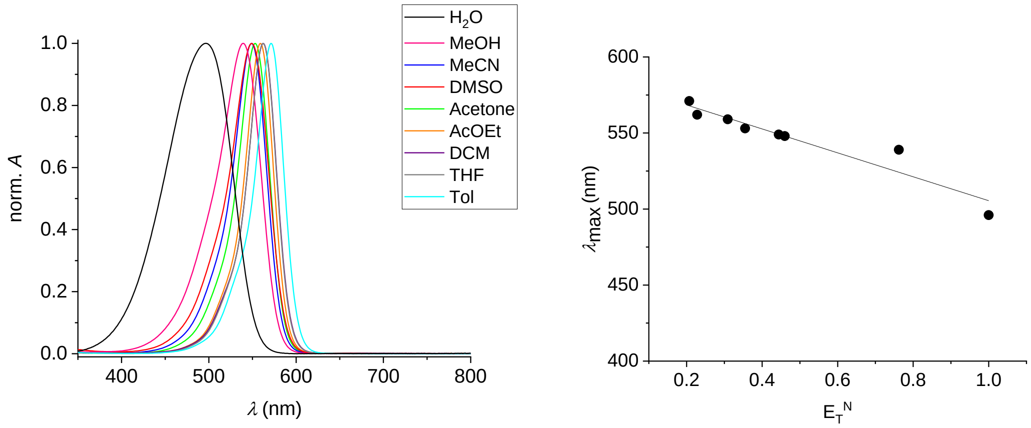

Figure S17. Solvatochromic shift analysis of DASA **1** in solvents (left) of varying polarity using the Dimroth-Reichardt $E_T^N(30)$ scale (right). Slope = –79 nm ($R^2 = 0.9048$).

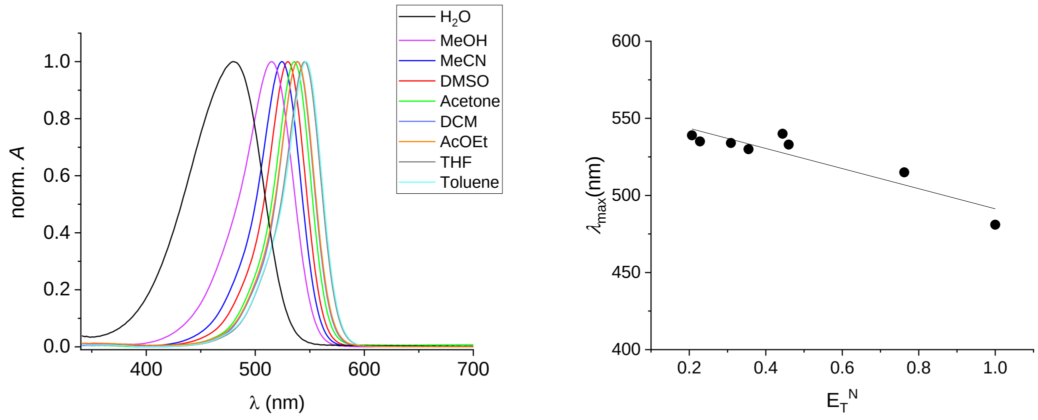

Figure S18. Solvatochromic shift analysis of DASA **2** in solvents (left) of varying polarity using the Dimroth-Reichardt $E_T^N(30)$ scale (right). Slope = –65 nm ($R^2 = 0.8357$).

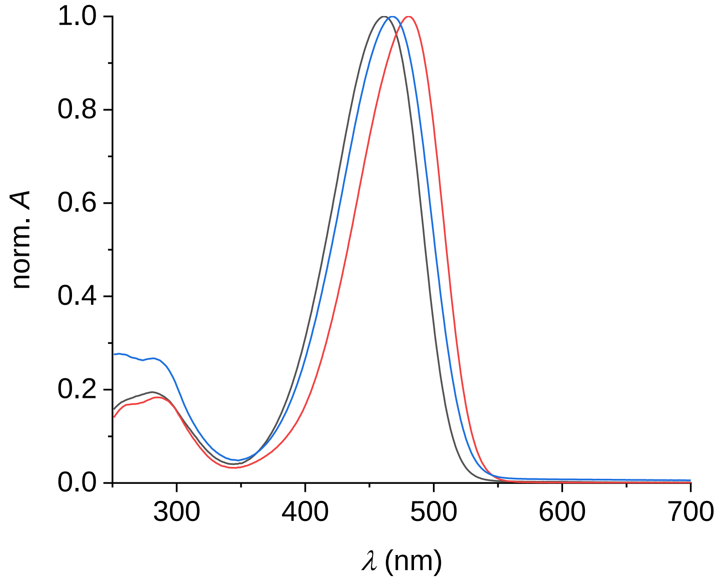

Figure S19. Normalized absorption spectra of free DASA **1** (red line) in its colored linear form and in the presence of 1 equivalent CB7 (black line) or CB8 (blue line).

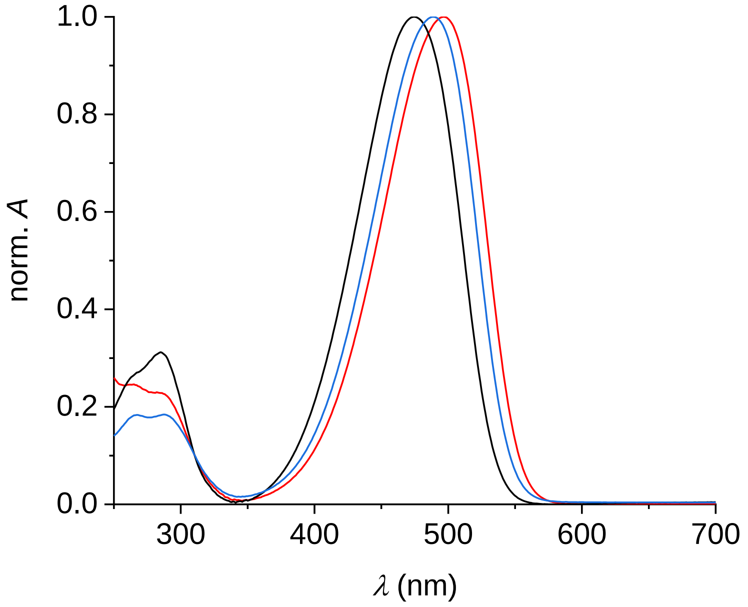

Figure S20. Normalized absorption spectra of free DASA **2** (red line) in its colored linear form and in the presence of 1 equivalent CB7 (black line) or CB8 (blue line).

6. Fluorescence emission of DASA 1 and DASA 2

Table S1. Photophysical properties of DASA **1** and DASA **2** in water; stabilized by CB7 or CB8.

	$\lambda_{max.abs}$ (nm)	$\Delta\lambda$ (nm)[a]	$\lambda_{max.f}$ (nm)	τ_f (ns)[b]	Φ_{fluo}
**1**@CB7	473	–25	537	0.91	<0.01
**1**@CB8	489	–9	568	1.43	0.03
**2**@CB7	463	–18	529	0.95	<0.01
**2**@CB8	473	–8	540	1.14	0.02

[a] Spectral shift of the dye upon addition of 1 equiv. CB7 or CB8. [b] Intensity-averaged lifetime.

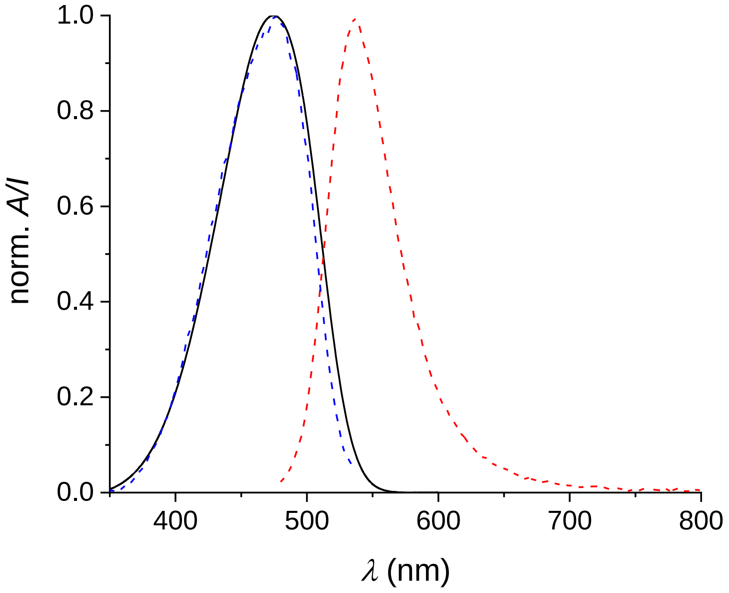

Figure S21. UV/vis absorption spectrum (black line), emission spectrum (λ_{exc} = 473 nm, red line), and excitation spectrum (blue line) of DASA **1** 20 µM in the presence of 1 equivalent of CB7 in water.

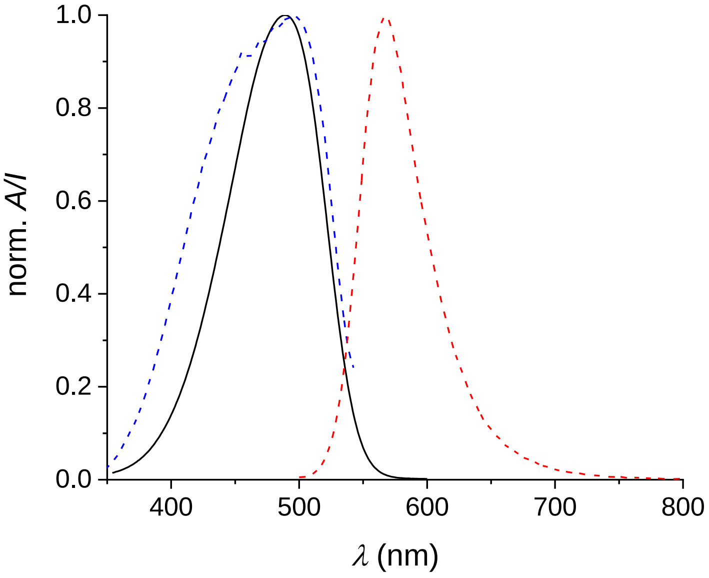

Figure S22. UV/vis absorption spectrum (black line), emission spectrum (λ_{exc} = 489 nm, red line), and excitation spectrum (blue line) of DASA **1** 20 µM in the presence of 1 equivalent of CB8 in water.

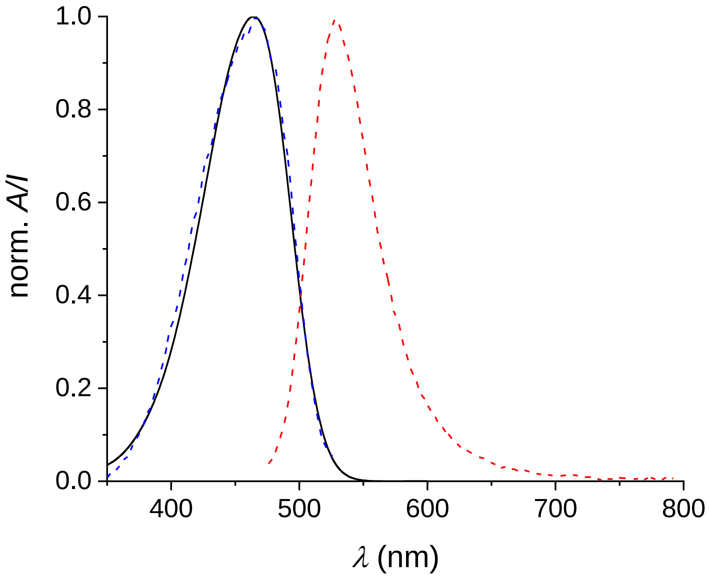

Figure S23. UV/vis absorption spectrum (black line), emission spectrum (λ_{exc} = 460 nm, red line), and excitation spectrum (blue line) of DASA **2** 20 µM in the presence of 1 equivalent of CB7 in water.

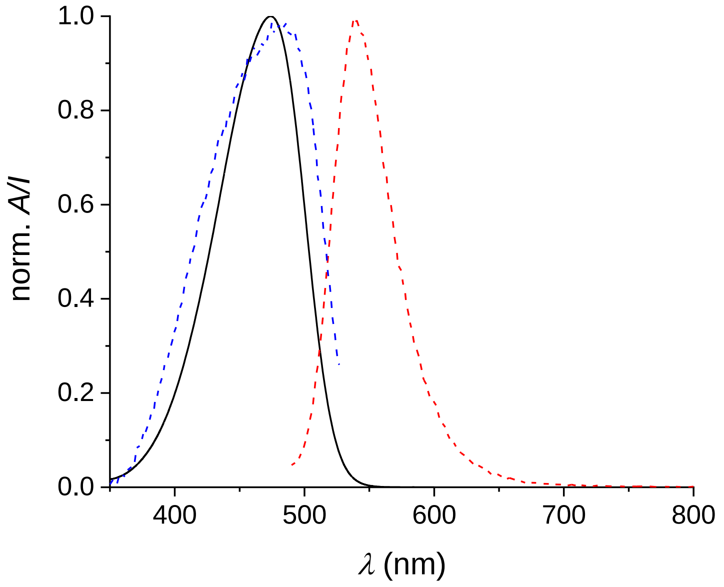

Figure S24. UV/vis absorption spectrum (black line), emission spectrum (λ_{exc} = 475 nm, red line), and excitation spectrum (blue line) of DASA **2** 20 µM in the presence of 1 equivalent of CB8 in water.

7. “Dark switching” and photoswitching of DASA 1 and DASA 2 – additional data

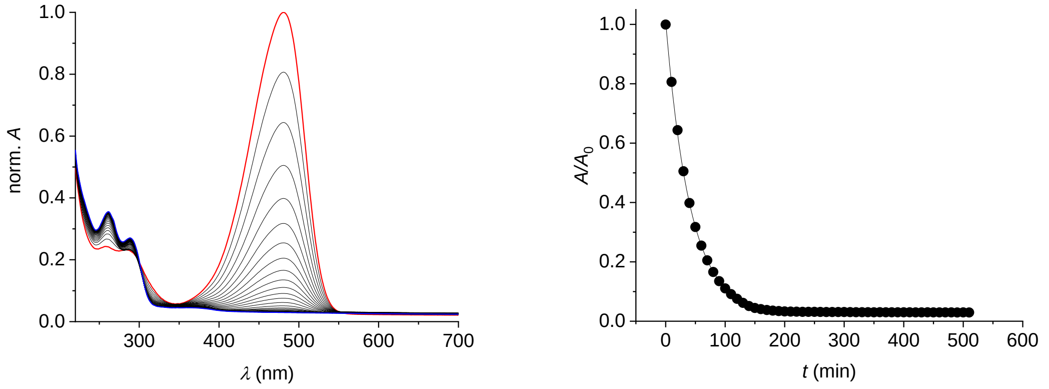

Figure S25. “Dark switching” of DASA **2** in water (10% THF), followed by UV/vis spectroscopy. Left: Spectral evolution (25 µM). Right: Kinetic decay (monitored at 482 nm) of the colored linear form.

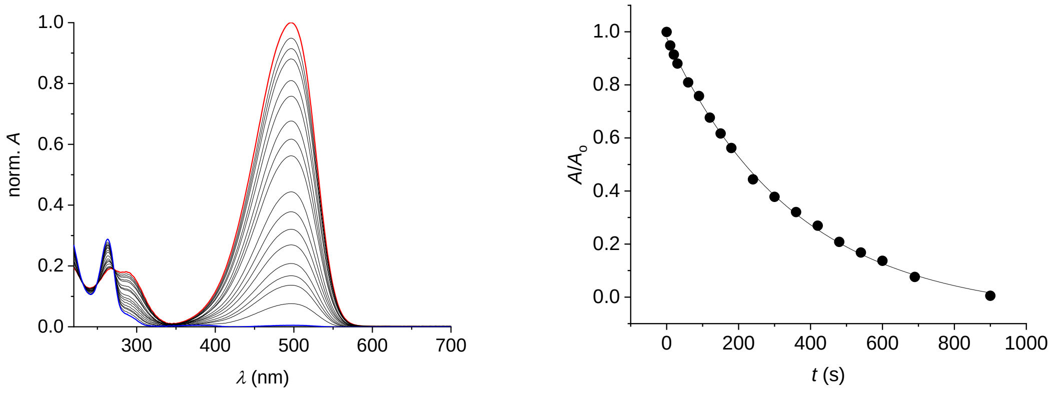

Figure S26. Ring closing of DASA **1** in water (10% THF) upon light irradiation at >455 nm, followed by UV/vis spectroscopy. Left: Spectral evolution (25 µM). Right: Kinetic decay (monitored at 495 nm) of the colored linear form.

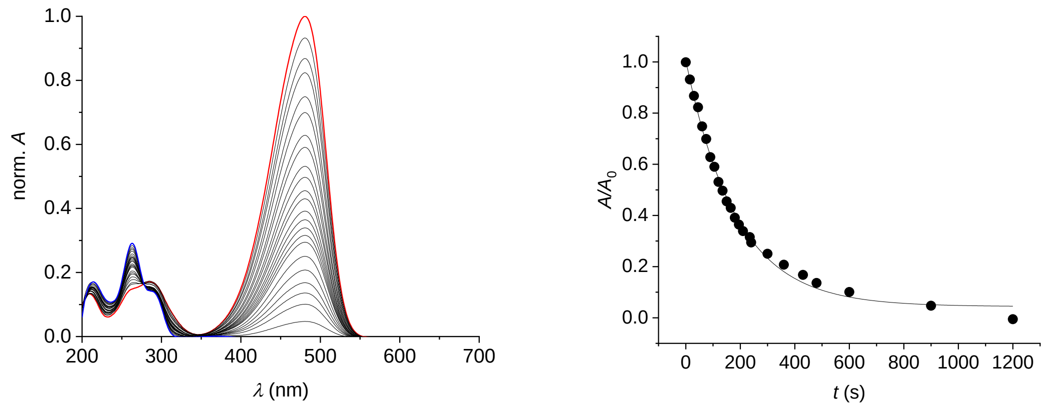

Figure S27. Ring closing of DASA **2** in water (10% THF) upon light irradiation at >455 nm, followed by UV/vis spectroscopy. Left: Spectral evolution (25 µM). Right: Kinetic decay (monitored at 482 nm) of the colored linear form.

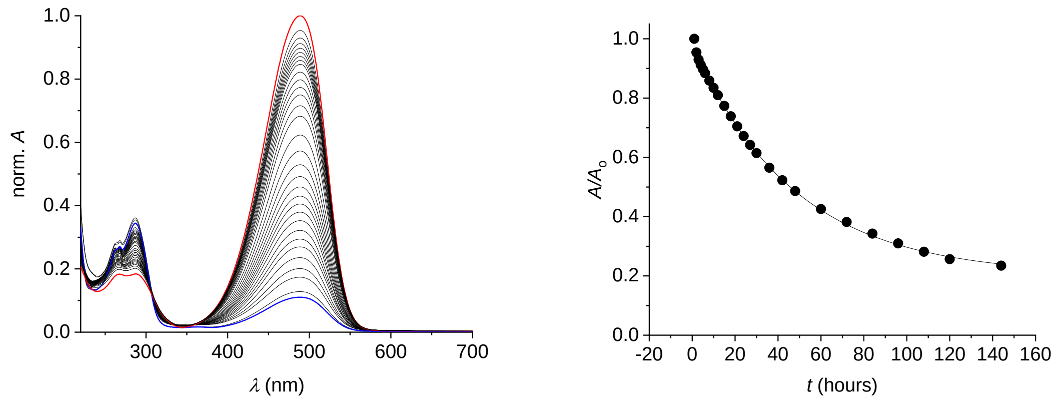

Figure S28. "Dark switching" of DASA **1**@CB8, followed by UV/Vis absorption spectroscopy. Left: Spectral evolution in the presence of 1 equivalent CB8 (20 µM). Right: Kinetic decay (monitored at 487 nm) of the colored linear form.

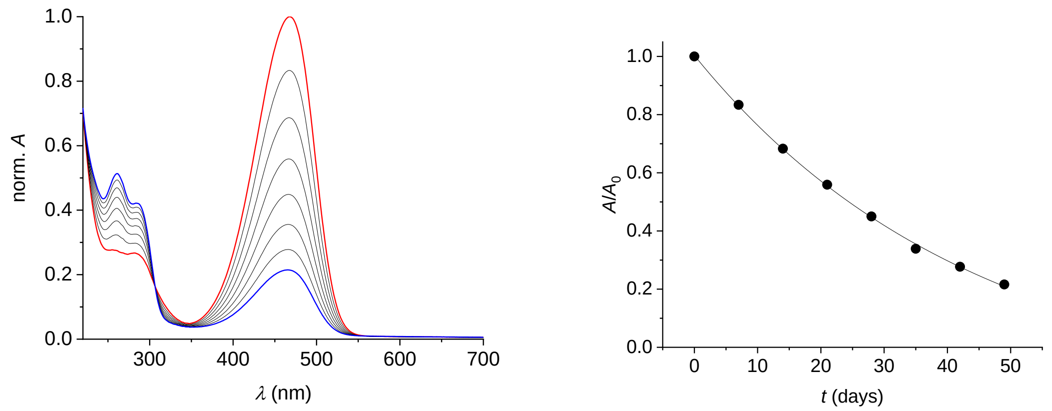

Figure S29. “Dark switching” of DASA **2**@CB7, followed by UV/vis absorption spectroscopy. Left: Spectral evolution in the presence of 1 equivalent CB7 (25 µM). Right: Kinetic decay (monitored at 461 nm) of the colored linear form.

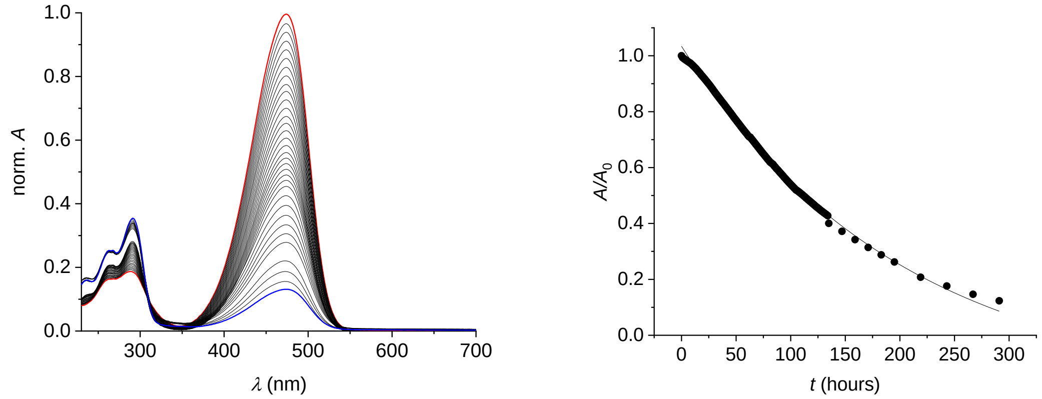

Figure S30. “Dark switching” of DASA **2**@CB8, followed by UV/vis absorption spectroscopy. Left: Spectral evolution in the presence of 1 equivalent CB8 (20 µM). Right: Kinetic decay (monitored at 473 nm) of the colored linear form.

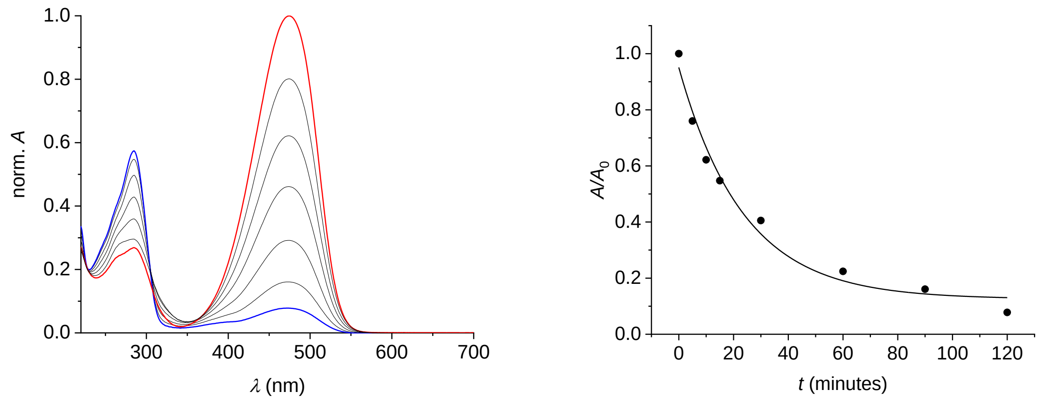

Figure S31. Ring closing of **1**@CB7 in water upon light irradiation at >455 nm, followed by UV/vis spectroscopy. Left: Spectral evolution in the presence of 1 equivalent CB7 (25 µM). Right: Kinetic decay (monitored at 474 nm) of the colored linear form.

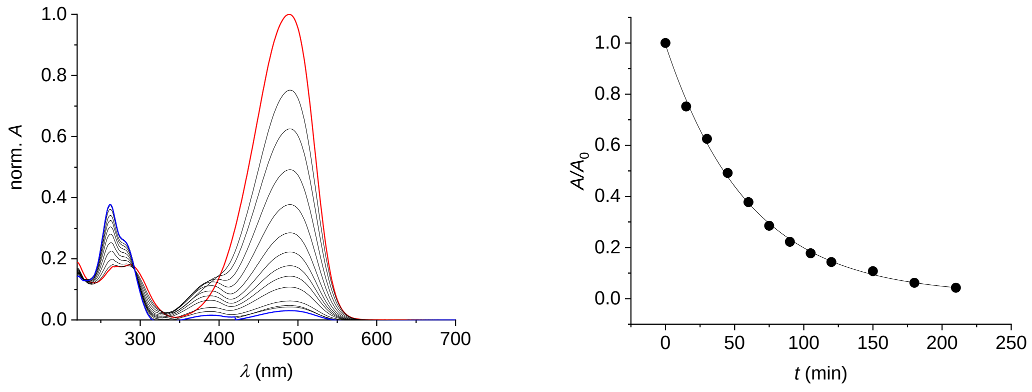

Figure S32. Ring closing of **1**@CB8 in water upon light irradiation at >455 nm, followed by UV/vis spectroscopy. Left: Spectral evolution in the presence of 1 equivalent CB8 (20 µM). Right: Kinetic decay (monitored at 487 nm) of the colored linear form.

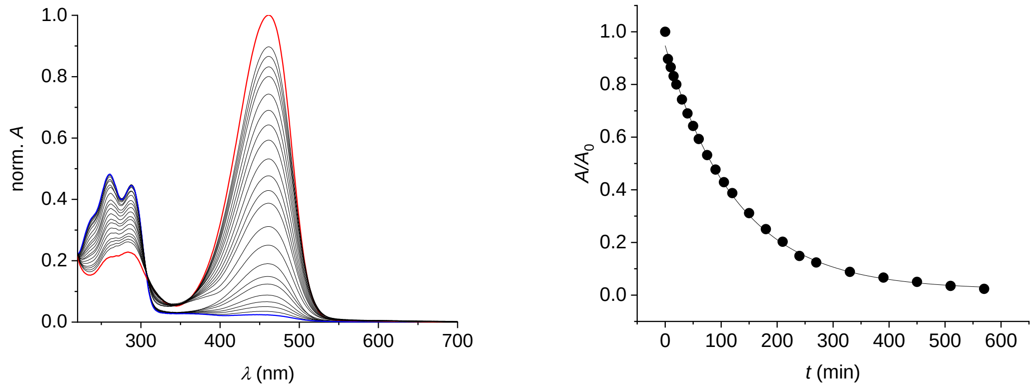

Figure S33. Ring closing of **2**@CB7 in water upon light irradiation at >455 nm, followed by UV/vis spectroscopy. Left: Spectral evolution in the presence of 1 equivalent CB7 (25 μM). Right: Kinetic decay (monitored at 461 nm) of the colored linear form.

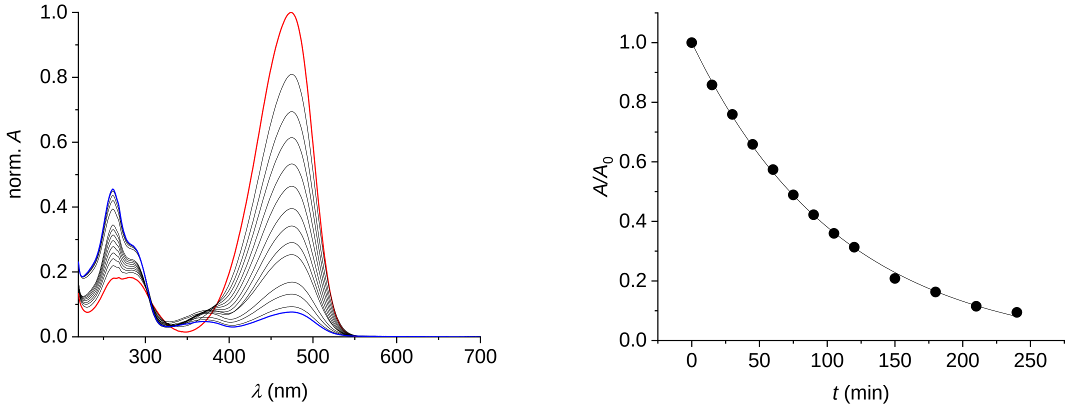

Figure S34. Ring closing of **2**@CB8 in water upon light irradiation at >455 nm, followed by UV/vis spectroscopy. Left: Spectral evolution in the presence of 1 equivalent CB8 (20 μM). Right: Kinetic decay (monitored at 473 nm) of the colored linear form.

8. Reversible photoswitching of DASA 2 in the presence of CB7 and CB8 in water

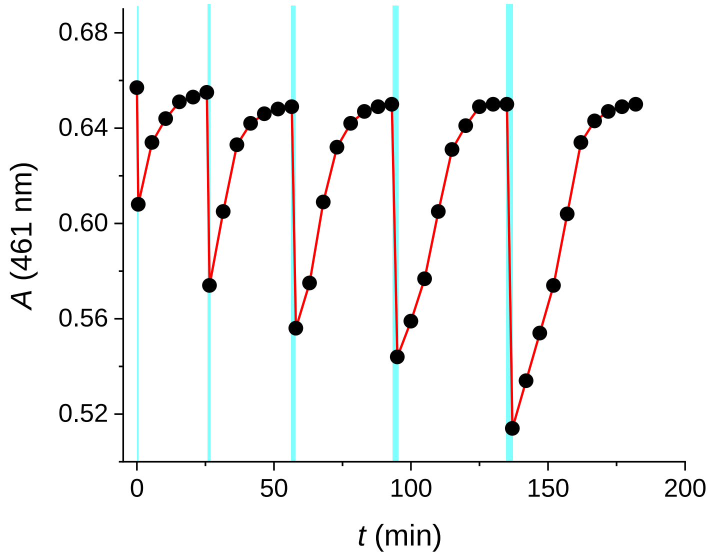

Figure S35. Switching cycles of DASA **2** (20 µM) in the presence of 1 equivalent CB7 in water. Irradiation at > 455 nm for 0.5, 1, 1.5, 2 and 2.5 min (cyan bars) and ring opening at 55 ºC.

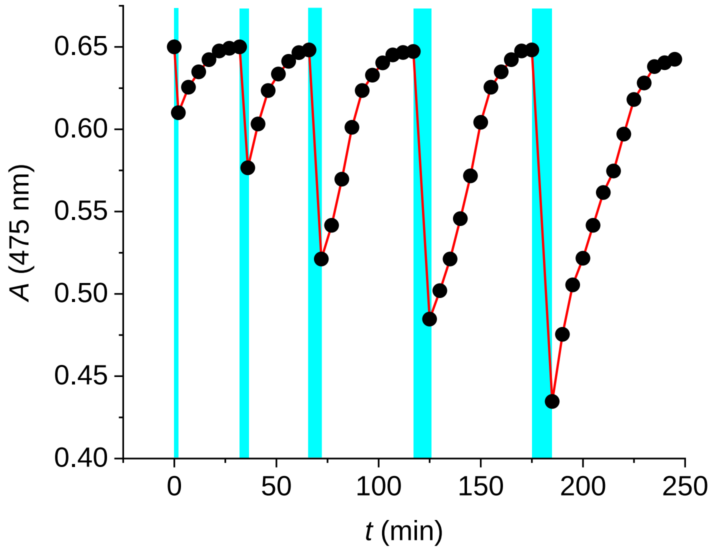

Figure S36. Switching cycles of DASA **2** (20 µM) in the presence of 1 equivalent CB8 in water. Irradiation at > 455 nm for 2, 4, 6, 8 and 10 min (cyan bars) and ring opening at 55 ºC.

9. Photoswitching of DASA 1 and DASA 2 in toluene

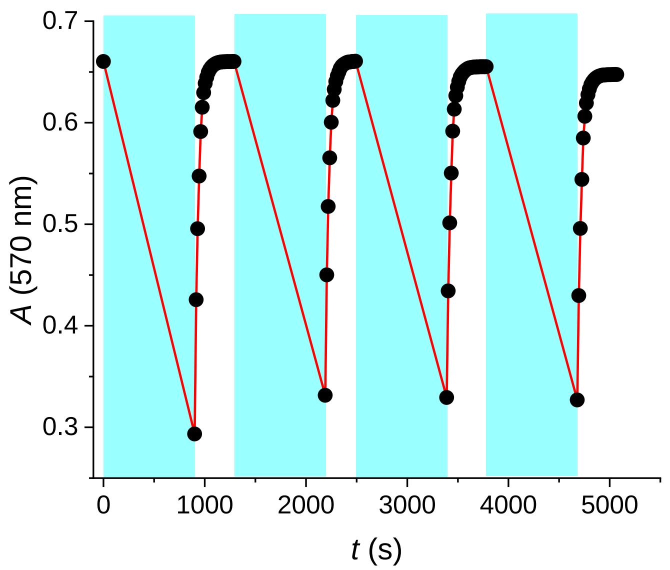

Figure S37. Switching cycles of DASA **1** in toluene (6 µM). Irradiation at > 455 nm for 15 minutes (cyan bars) and ring opening at room temperature. Rate constant for ring opening: k = 0.027 s^{-1}.

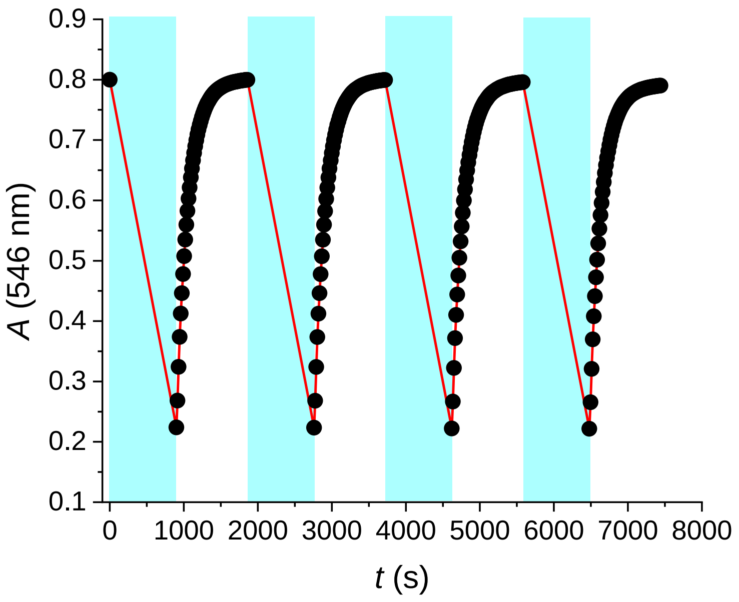

Figure S38. Switching cycles of DASA **2** in toluene (7.5 µM). Irradiation at > 455 nm for 15 minutes (cyan bars) and ring opening at room temperature. Rate constant for ring opening: k = 0.0065 s^{-1}.

10. Theoretical calculations

The adamantylated DASA open form was built in its EEZ configuration (A isomer) and the closed form (B isomer) was modeled in its zwitterionic state. All structures were initially optimized at the GFN2-xTB level[5, 6] using the xtb (6.6.1) program with the ALPB implicit water model.[7] Low-lying conformers for both isomers were then located with CREST (3.0.2),[8] employing the default iMTD-GC sampling. The resulting geometries were refined at the DFT level, using the composite meta-GGA r^2SCAN-3c method[9] (def2-mTZVPP basis, D4 dispersion) and the CPCM solvation model for water[10] in ORCA (6.0.1).[11] Vibrational frequencies of these DFT geometries were computed at the GFN2-xTB level via the biased-Hessian approach to confirm minima and obtain thermostatistical corrections (298.15 K, 1 atm).[12]

To identify the preferred CB7/CB8 binding mode for both open and closed DASA, the host (H)–guest (G) complexes (HG) were assembled in two ways: with the CB macrocycle threaded over the aminoadamantane moiety or over the barbituric acid/Meldrum's acid site. These assemblies were optimized at the GFN2-xTB level[5, 6] and then subjected to a minima-hopping conformer search (xtb --metaopt, with save=1000, kpush=0.1 Eh, α=1.0 Bohr^{-1}).[13] Resulting structures were clustered using CREST's CREGEN routine within a 6 kcal mol^{-1} energy window. The cluster ensemble was re-optimized at GFN2-xTB level[5, 6] with a tightened convergence threshold (1×10^{-7} Eh) and the vibrational frequencies were computed to confirm true minima. The three lowest-energy conformers for each binding motif were subsequently re-optimized at the DFT level [r^2SCAN-3c,[9] CPCM(water)[10]]. Biased-Hessian GFN2-xTB frequencies (single-point Hessian on the DFT geometries) provided thermochemical corrections ($CORR^{GFN2}$). The total free energies of the lowest-energy conformers were obtained as

$$G^{\mathrm{corr}} = E^{\mathrm{DFT}} + \mathrm{CORR}^{\mathrm{GFN2}} \quad (1)$$

and relative binding free energies by

$$\Delta G^{\mathrm{corr}} = G_{\mathrm{HG}}{}^{\mathrm{corr}} - G_{\mathrm{G}}{}^{\mathrm{corr}} - G_{\mathrm{H}}{}^{\mathrm{corr}} \quad (2)$$

Relative electronic binding energies were computed analogously from DFT total energies:

$$\Delta E^{\mathrm{DFT}} = E_{\mathrm{HG}}{}^{\mathrm{DFT}} - E_{\mathrm{G}}{}^{\mathrm{DFT}} - E_{\mathrm{H}}{}^{\mathrm{DFT}} \quad (3)$$

Table S2 summarizes the relative electronic binding energies (ΔE^{DFT}) and relative binding free energies (ΔG^{corr}) for the open and closed forms of the DASA dyes complexed with CB7 or CB8 at either the aminoadamantane or the barbituric acid/Meldrum's acid site.

Table S2. Relative binding energies and binding free energies of DASA **1** and DASA **2** by CB7 and CB8; in kcal mol^{-1}.

	ΔE^{DFT}	$\Delta G^{corr\ a}$	ΔE^{DFT}	$\Delta G^{corr\ a}$
	CB7@DASA		CB7@aminoadamantane	
Linear **1**	–16.9	+3.5	–29.0	–10.0
Closed **1**[b]	–6.2	+13.0	–31.5	–11.8
Linear **2**	–17.3	+2.0	–28.7	–9.9
Closed **2**[b]	–16.8	+3.4	–28.2	–9.4
	CB8@DASA		CB8@aminoadamantane	
Linear **1**	–19.8	–0.1	–22.8	–4.2
Closed **1**[b]	–23.7	–3.1	–20.2	–2.0
Linear **2**	–16.8	+2.6	–21.6	–3.7
Closed **2**[b]	–22.2	–3.4	–24.7	–7.1

[a] The binding free energies ΔG^{corr} indicate preferential complexation at the aminoadamantane site of DASA **1** and DASA **2** with CB7 and CB8 hosts. This statement refers to both the colored linear and closed form of the DASA, except for the complexation of the closed form of DASA **1** by CB8. For this case the aminoadamantane site and the DASA-related part seem to have similar binding probabilities. Furthermore the results show that CB7 is generally a stronger binder of the dyes than CB8. B The closed form was optimized as zwitterionic form B (see Scheme 1 in main text).

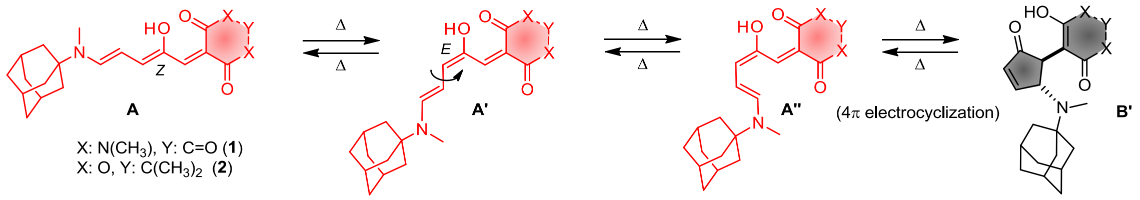

Scheme S1. "Dark switching" of DASA **1** and DASA **2**, according to the mechanistic picture reported in ref. 14.

To investigate the switching mechanism of the guests and of the CB7 complexes (binding at the aminoadamantane site), relaxed scans were performed starting from the

A isomer (or A@CB7 complex) at the GFN2-xTB level.[5, 6] Candidate transition-state (TS) structures (highest-energy points along the scanned coordinate) and product minima were optimized at the GFN2 level in ORCA[11] by invoking GFN2 via ORCA's xTB interface (! XTB2). Geometry optimizations used OPT for minima and OPTTS for TS structures with the ALPB implicit solvent model for water;[7] vibrational frequency calculations were used to confirm minima (no imaginary frequencies) and transition states (one imaginary frequency). The conformer searches followed the same minima-hopping protocol as used for the host–guest systems. During the TS conformer searches the dihedral angles or bond lengths involved in the reaction coordinate were constrained (force constant = 0.7), and constraints were removed for the final TS optimizations. The lowest-energy structures were re-optimized at the GFN2 level and their vibrational spectra and intrinsic reaction coordinates (for transition states) were recomputed to confirm the nature of the stationary points and calculate thermochemical corrections, using ORCA's default quasi-rigid-rotor-harmonic-oscillator (qRRHO). For each minimum and transition state the three lowest-energy structures of guests and CB7 complexes were further refined at the DFT level.

Because r^2SCAN-3c gives excellent reaction and conformational energies, but can be less reliable for accurate barrier heights,[9, 15] we refined minima and transition-state geometries with the hybrid functional M06-2X.[16-18] The minima and transition-state structures of the reaction trajectory of DASA dyes alone were refined at M06-2X/def2-SVP level,[16-18] using an enlarged integration grid (DefGrid3), the D3 dispersion correction with zero damping [D3(0)], the geometrical counterpoise (gCP) correction, and the CPCM solvent model (water).[10] For the host–guest complexes, where hybrid DFT is computationally demanding, a two-layer ONIOM approach in ORCA[11] was used: the guest was treated as the high-level region and CB7 as the low-

level region (no link atoms). Default ORCA settings were used for the subtractive ONIOM scheme with electrostatic embedding. In the QM/xTB multiscale scheme the low-level region was treated with GFN2-xTB[5, 6] and for the high-level region M06-2X/def2-SVP[16-18] [DefGrid3, D3(0)] and ALPB(water) solvation[7] was used. Final single-point energies on the refined complex geometries were computed at the full DFT level M06-2X/def2-SVP[16-18] with D3(0), CPCM(water),[10] and gCP to reduce basis-set superposition error. The lowest-lying conformer electronic energies [M06-2X/def2-SVP,[16-18] D3(0), CPCM(water),[10] gCP], together with thermochemical corrections obtained from GFN2-xTB[5, 6] frequency calculations, were combined to yield Gibbs free energies (G^{corr}) of minima and transition states for construction of the reaction surfaces of both guests and their CB7 complexes (Table S3 and Figure S39-S42).

Table S3. Free activation energies for the forward and backward thermal isomerization of DASA **1** and DASA **2** in the absence and presence of CB7 or CB8 in water.

Step	$\Delta G^{\ddagger}$ (kcal·mol^{-1})[a]			
	**1**	**1**@CB7	**2**	**2**@CB7
forward process (linear→closed)				
A→A’	19.6	20.1	19.8	19.8
A’→A”	13.9	23.0	16.0	27.4
A”→B’	17.7	19.5	18.6	26.0
backward process (closed→linear)				
B`→A”	12.4	14.7	13.6	21.9
A”→A’	9.8	10.6	11.8	14.2
A’→A	14.5	15.0	14.8	14.4

[a] The activation free energies $\Delta G^{\ddagger}$ clearly indicate that the barriers for the forward process are considerably raised for DASA **1** and DASA **2** when complexed by CB7 at the adamantane moiety. This statement applies especially to the formation of the A” intermediate by bond rotation (DASA **1** and DASA **2**) and the ring-closing by 4π electrocyclization (A”→B’ for DASA **2**). This explains the slowing down of the “dark switching”, resulting in the kinetic stabilization of the colored linear form. Furthermore, the significant stabilization of the closed form of DASA **2** by CB7 (back isomerization is only observed upon application of heat) finds its reasoning in the increased $\Delta G^{\ddagger}$ of the B`→A” step. The situations where the increase of $\Delta G^{\ddagger}$ upon complexation with CB7 exceeds 3 kcal·mol^{-1} are color-marked.

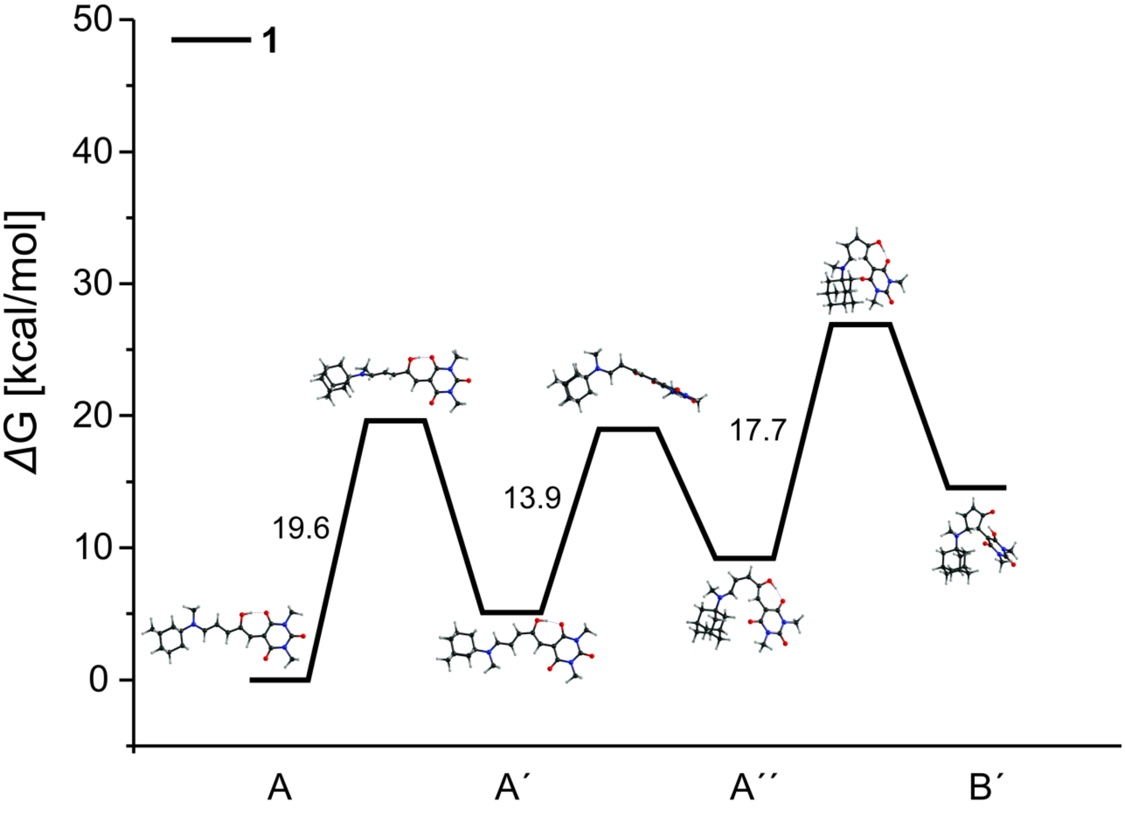

Figure S39. Energy profile for the thermal isomerization of DASA **1** in water. For the structures of the intermediates **A**, **A'**, **A''**, and **B'** see Scheme S1. The activation free energies for the forward thermal isomerization are indicated, along with the optimized structures of the intermediates and transition states.

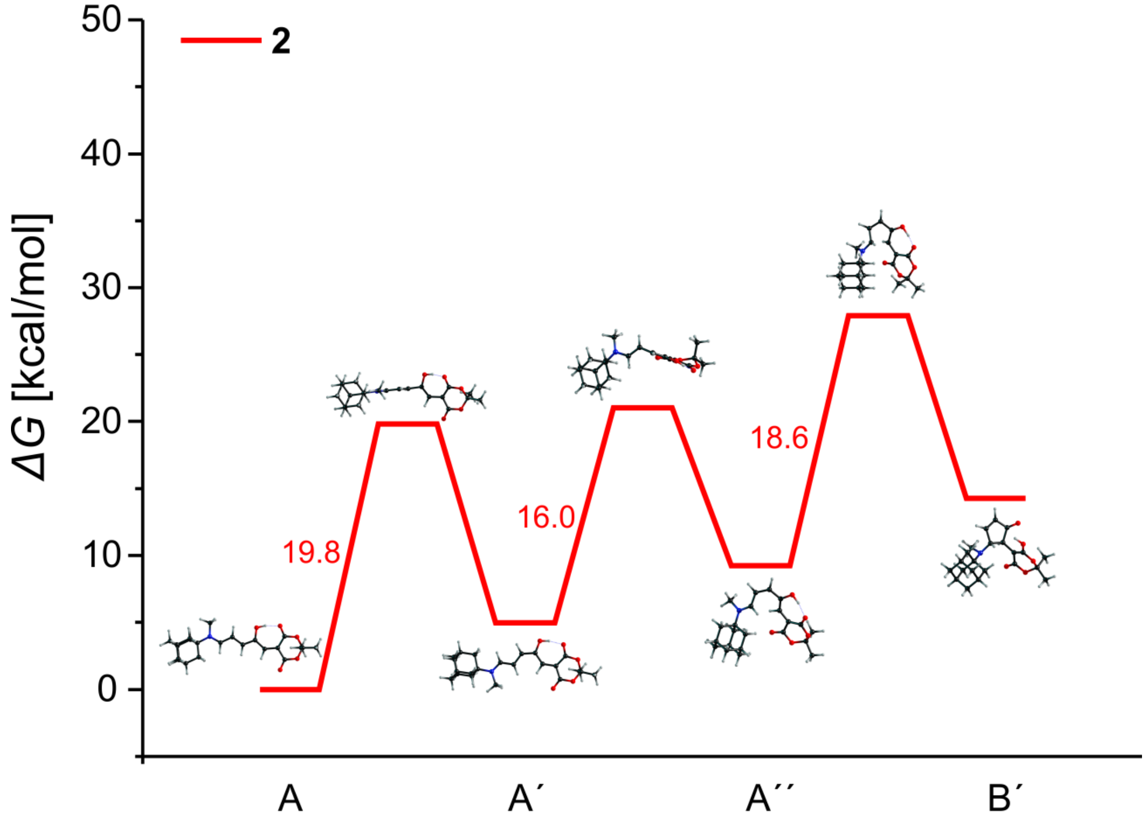

Figure S40. Energy profile for the thermal isomerization of DASA **2** in water. For the structures of the intermediates **A**, **A'**, **A''**, and **B'** see Scheme S1. The activation free energies for the forward thermal isomerization are indicated, along with the optimized structures of the intermediates and transition states.

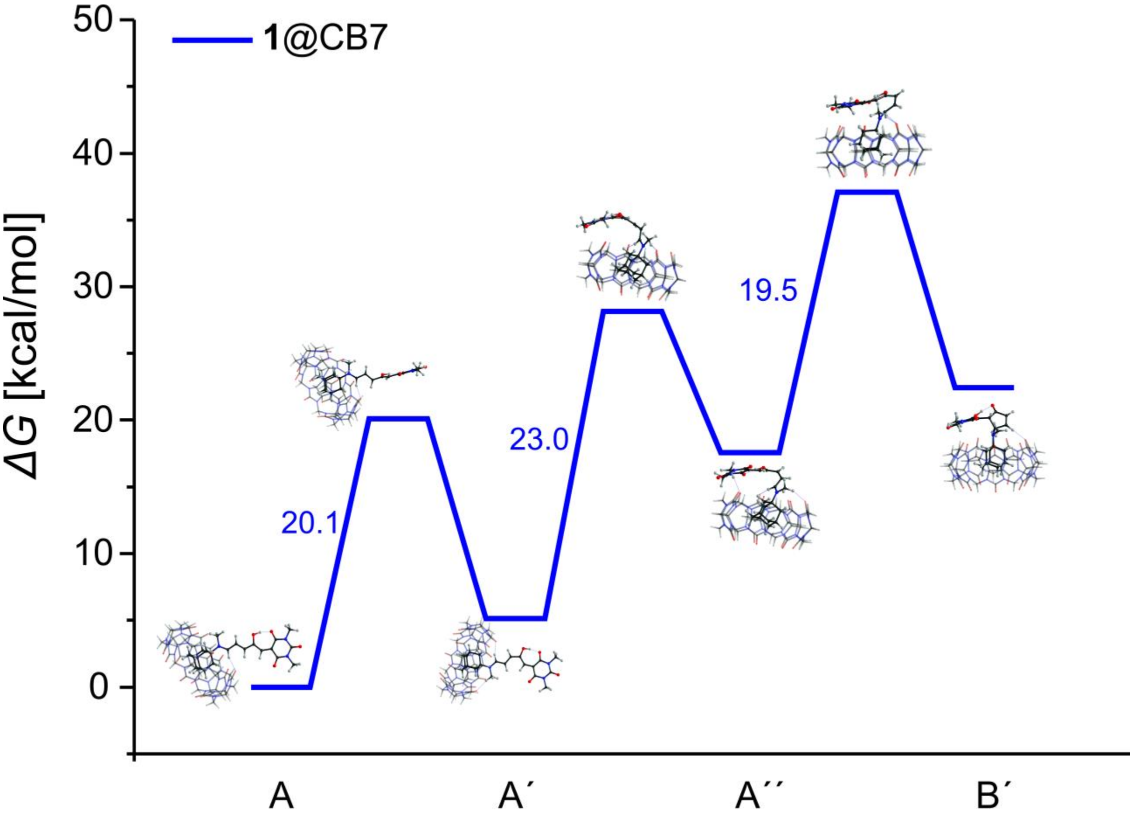

Figure S41. Energy profile for the thermal isomerization of DASA **1** complexed by CB7 in water. For the structures of the intermediates **A**, **A'**, **A''**, and **B'** see Scheme S1. The activation free energies for the forward thermal isomerization are indicated, along with the optimized structures of the intermediates and transition states.

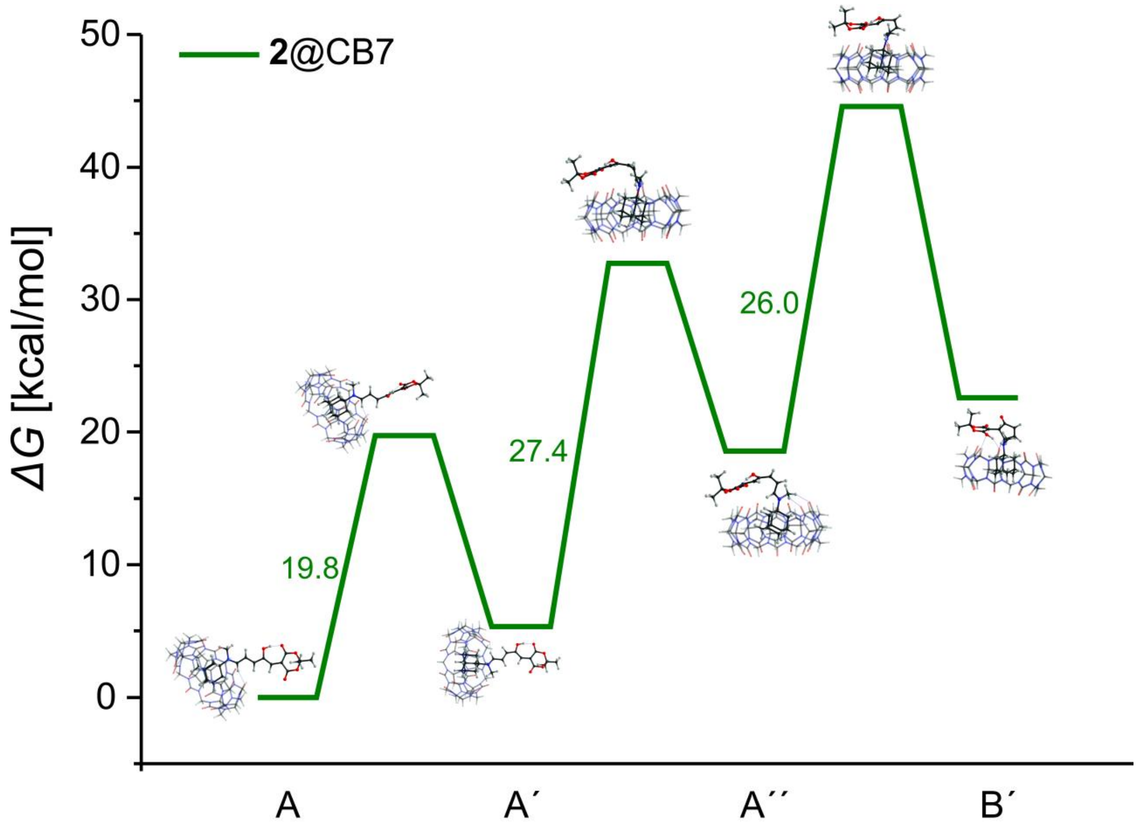

Figure S42. Energy profile for the thermal isomerization of DASA **2** complexed by CB7 in water. For the structures of the intermediates **A**, **A'**, **A''**, and **B'** see Scheme S1. The activation free energies for the forward thermal isomerization are indicated, along with the optimized structures of the intermediates and transition states.

Energy-decomposition analysis (EDA-NOCV) was performed in ORCA between guest (**1** and **2**) and CB7 as fragments to rationalize the larger reaction barriers observed for **2**@CB7. EDA was carried out at the M06-2X/def2-SVP level (D3ZERO, CPCM(water), gCP) using ORCA default settings for EDA-NOCV (see Figure S43).

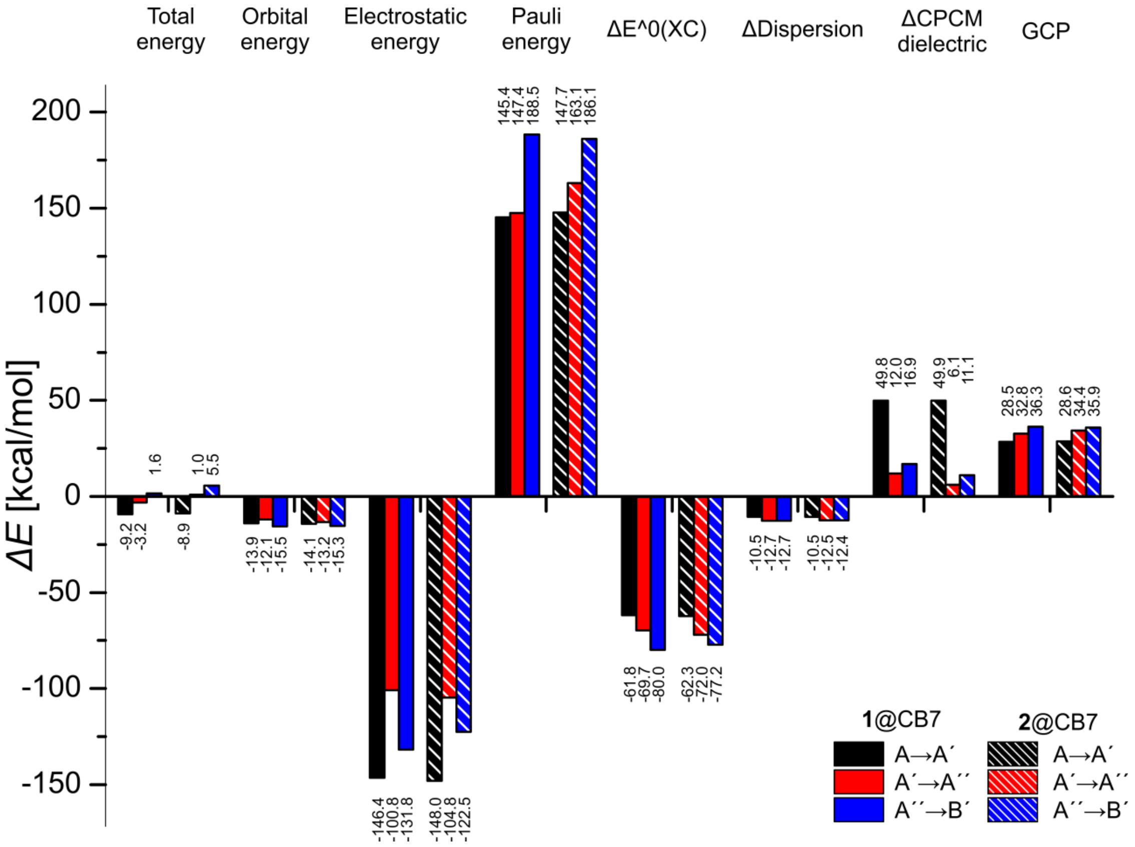

Figure S43. Energy decomposition analysis of the transition states (TS) of the thermal isomerization path of the supramolecular CB7 complexes of DASA **1** and DASA **2** in water. This analysis indicates that the main differences between the CB7-complexes of DASA **1** and DASA **2** are found for the steric (Pauli) repulsion in the TS of the A'→A" step and the electrostatic attraction in the TS of the A"→B' step. The comparably larger steric repulsion energy and weaker electrostatic attraction accounts for the higher activation barriers in the case of DASA **2**.

11. References

1. Kuhn, H. J.; Braslavsky, S. E.; Schmidt, R., Chemical Actinometry (IUPAC Technical Report). *Pure Appl. Chem.* **2004,** *76*, 2105‒2146.

2. Hatchard, C. G.; Parker, C. A.; Bowen, E. J., A New Sensitive Chemical Actinometer - II. Potassium Ferrioxalate as a Standard Chemical Actinometer. *Proc. Roy. Soc. Lond. A* **1956**, *235*, 518‒536.

3. Deb, M. L.; Bhuyan, P. J., Uncatalysed Knoevenagel Condensation in Aqueous Medium at Room Temperature. *Tetrahedron Lett.* **2005**, *46*, 6453‒6456.

4. Bigi, F.; Carloni, S.; Ferrari, L.; Maggi, R.; Mazzacani, A.; Sartori, G., Clean Synthesis in Water. Part 2: Uncatalysed Condensation Reaction of Meldrum's Acid and Aldehydes. *Tetrahedron Lett.* **2001**, *42*, 5203‒5205.

5. Bannwarth, C.; Ehlert, S.; Grimme, S., GFN2-xTB ‒ An Accurate and Broadly Parametrized Self-Consistent Tight-Binding Quantum Chemical Method with Multipole Electrostatics and Density-Dependent Dispersion Contributions. *J. Chem. Theory Comput.* **2019**, *15*, 1652‒1671.

6. Bannwarth, C.; Caldeweyher, E.; Ehlert, S.; Hansen, A.; Pracht, P.; Seibert, J.; Spicher, S.; Grimme, S., Extended Tight-Binding Quantum Chemistry Methods. *WIREs Comput. Mol. Sci.* **2020**, *11*, e1493.

7. Ehlert, S.; Stahn, M.; Spicher, S.; Grimme, S., Robust and Efficient Implicit Solvation Model for Fast Semiempirical Methods. *J. Chem. Theory Comput.* **2021**, *17*, 4250‒4261.

8. Pracht, P.; Bohle, F.; Grimme, S., Automated Exploration of the Low-Energy Chemical Space with Fast Quantum Chemical Methods. *Phys. Chem. Chem. Phys.* **2020**, *22*, 7169‒7192.

9. Grimme, S.; Hansen, A.; Ehlert, S.; Mewes, J.-M., r^{2}SCAN-3c: A "Swiss Army Knife" Composite Electronic-Structure Method. *J. Chem. Phys.* **2021**, *154*, 064103.

10. Garcia-Ratés, M.; Neese, F., Effect of the Solute Cavity on the Solvation Energy and its Derivatives within the Framework of the Gaussian Charge Scheme. *J. Comput. Chem.* **2020**, *41*, 922–939.

11. Neese, F., Software Update: The ORCA Program System, Version 5.0. *WIREs Comput. Mol. Sci.* **2022**, *12*, e1606.

12. Spicher, S.; Grimme, S., Single-Point Hessian Calculations for Improved Vibrational Frequencies and Rigid-Rotor-Harmonic-Oscillator Thermodynamics. *J. Chem. Theory Comput.* **2021**, *17*, 1701–1714.

13. Grimme, S., Exploration of Chemical Compound, Conformer, and Reaction Space with Meta-Dynamics Simulations Based on Tight-Binding Quantum Chemical Calculations. *J. Chem. Theory Comput.* **2019**, *15*, 2847–2862.

14. Wang, D.; Zhao, L.; Zhao, H.; Wu, J.; Wagner, M.; Sun, W.; Liu, X.; Miao, M.; Zheng, Y., Inducing Molecular Isomerization Assisted by Water. *Commun. Chem.* **2019**, *2*, 118.

15. Gasevic, T.; Stückrath, J. B.; Grimme, S.; Bursch, M., Optimization of the r^{2}SCAN-3c Composite Electronic-Structure Method for Use with Slater-Type Orbital Basis Sets. *J. Phys. Chem. A* **2022**, *126*, 3826–3838.

16. Zhao, Y.; Truhlar, D. G., The M06 Suite of Density Functionals for Main Group Thermochemistry, Thermochemical Kinetics, Noncovalent Interactions, Excited States, and Transition Elements: Two New Functionals and Systematic Testing of Four M06-Class Functionals and 12 Other Functionals. *Theor. Chem. Account* **2008**, *120*, 215–241.

17. Goerigk, L.; Grimme, S., A Thorough Benchmark of Density Functional Methods for General Main Group Thermochemistry, Kinetics, and Noncovalent Interactions. *Phys. Chem. Chem. Phys.* **2011**, *13*, 6670–6688.

18. Prasad, V. K.; Pei, Z.; Edelmann, S.; Otero-de-la-Roza, A.; DiLabio, G. A., BH9, a New Comprehensive Benchmark Data Set for Barrier Heights and Reaction Energies: Assessment of Density Functional Approximations and Basis Set Incompleteness Potentials. *J. Chem. Theory Comput.* **2022**, *18*, 151–166.
